# Plant growth promoting potential of urea doped calcium phosphate nanoparticles in finger millet (*Eleusine coracana* (L.) Gaertn.) under drought stress

**DOI:** 10.3389/fpls.2023.1137002

**Published:** 2023-05-15

**Authors:** Dhruv Mishra, Manoj Kumar Chitara, Viabhav Kumar Upadhayay, Jagat Pal Singh, Preeti Chaturvedi

**Affiliations:** ^1^ Department of Biological Sciences, College of Basic Sciences and Humanities, G.B. Pant University of Agriculture and Technology, Pantnagar, Uttarakhand (U.K.), India; ^2^ Department of Plant Pathology, College of Agriculture, G.B. Pant University of Agriculture and Technology, Pantnagar, Uttarakhand, India; ^3^ Department of Microbiology, College of Basic Sciences & Humanities, Dr. Rajendra Prasad Central Agricultural University, Samastipur, Bihar, India; ^4^ Department of Physics, College of Basic Sciences and Humanities, G. B. Pant University of Agriculture and Technology, Pantnagar, India

**Keywords:** urea, finger millet, drought, plant growth promotion, calcium phosphate (Ca-P), nanoparticles

## Abstract

Drought is a leading threat that impinges on plant growth and productivity. Nanotechnology is considered an adequate tool for resolving various environmental issues by offering avant-garde and pragmatic solutions. Using nutrients in the nano-scale including CaP-U NPs is a novel fertilization strategy for crops. The present study was conducted to develop and utilize environment-friendly urea nanoparticles (NPs) based nano-fertilizers as a crop nutrient. The high solubility of urea molecules was controlled by integrating them with a matrix of calcium phosphate nanoparticles (CaP NPs). CaP NPs contain high phosphorous and outstanding biocompatibility. Scanning electron microscopy (FE-SEM), transmission electron microscopy (TEM) and X-ray diffraction analysis (XRD) were used to characterize the fabricated NPs. FE-SEM determined no areas of phase separation in urea and calcium phosphate, indicating the successful formation of an encapsulated nanocomposite between the two nano matrices. TEM examination confirmed a fiber-like structure of CaP-U NPs with 15 to 50 nm diameter and 100 to 200 nm length. The synthesized CaP-U NPs and bulk urea (0.0, 0.1% and 0.5%) were applied by foliar sprays at an interval of 15 days on pre-sowed VL-379 variety of finger millet (*Eleusine coracana* (L.) Gaertn.), under irrigated and drought conditions. The application of the CaP-U NPs significantly enhanced different plant growth attributes such as shoot length (29.4 & 41%), root length (46.4 & 51%), shoot fresh (33.6 & 55.8%) and dry weight (63 & 59.1%), and root fresh (57 & 61%) and dry weight (78 & 80.7%), improved pigment system (chlorophyll) and activated plant defense enzymes such as proline (35.4%), superoxide dismutase (47.7%), guaiacol peroxidase (30.2%), ascorbate peroxidase (70%) under both irrigated and drought conditions. Superimposition of five treatment combinations on drought suggested that CaP-U NPs at 0.5 followed by 0.1% provided the highest growth indices and defense-related enzymes, which were significantly different. Overall, our findings suggested that synthesized CaP-U NPs treatment of finger millet seeds improved plant growth and enzymatic regulation, particularly more in drought conditions providing insight into the strategy for not only finger millet but probably for other commercial cereals crops which suffer from fluctuating environmental conditions.

## Graphical Abstract

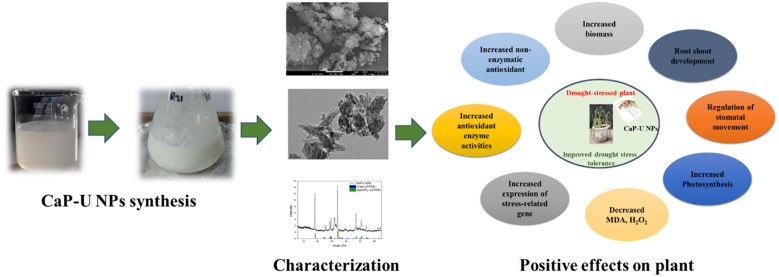

## 1 Introduction

Drought is one of the important abiotic stresses, where plants constantly do not receive sufficient rainfall required to complete their metabolic activities (Zhang et al., 2022). The unavailability of water reduces 9-10% of the total crop productivity worldwide (Lesk et al., 2016). Monsoon rainfall has decreased by roughly 6% from 1951 to 2015 as per the climate change assessment report by the Ministry of Earth and Science (Krishnan et al., 2020). Low rainfall makes the dry land areas more vulnerable to runoff losses leading to drought proneness. A study from South Africa during 2017-2021 revealed that 25000 agricultural sector-based workers lost their jobs due to the adverse impact of drought on the economy (Orimoloye et al., 2022).

Drought impacts seed germination, the number of tillers, spikes, grain per plant, grain weight, plant stand and grain yield (Oumarou Abdoulaye et al., 2019; Ning et al., 2020; Gui et al., 2021a; Gui et al., 2021b; Sheteiwy et al., 2021a; Sheteiwy et al., 2021b; Ben-Jabeur et al., 2022; Sial et al., 2022; Sheteiwy et al., 2022). The drought also impairs the nutrient absorption ability of plants from the upper soil horizon (Abdelaal et al., 2021). It also modulates the physiological processes of the plants such as photosynthesis, respiration, leaf water potential, mineral absorption, circadian rhythm and hormone regulation etc. (Seleiman et al., 2021; Wang et al., 2021; Ghani et al., 2022; Hemati et al., 2022). Drought stress increases the production of reactive oxygen species (ROS) such as superoxide (O_2_^-^), singlet oxygen (O_2_), hydrogen peroxide (H_2_O_2_), and hydroxyl radicals (OH^-^) to levels that are frequently greater than the plant’s scavenging capability (Chitara et al., 2017; Kumari et al., 2021; Sachdev et al., 2021). ROS damages cells and cellular components, impair physiological and biochemical processes and can even cause plant death (Xie et al., 2019). Owing to ROS-induced oxidative stress increased electrolyte leakage followed by lipid peroxidation and plasmalemma damage. Lipid peroxidation causes the breakdown of polyunsaturated lipids in ketones and malondialdehyde (MDA) (Ju et al., 2018). Excessive ROS generation causes site-specific amino acid modification, peptide chain fragmentation, changed electric charge and enhanced protein proteolysis. ROS causes deoxyribose oxidation, strand breaks, nucleotide loss, a variety of changes to the bases of the nucleotides, and DNA-protein crosslinks (Sharma et al., 2019; Ahmed and Lingner, 2020).

Finger millet (*Eleusine coracana* (L.) Gaertn) is a millet crop widely produced in tropical and subtropical areas of Asia and Africa. It contains a very high amount of calcium (344 mg/100 g). The millet seed coat is an edible component of the kernel and has a high concentration of dietary fiber, phytochemicals such as polyphenols (0.2–3.0%) and high gallic acid (Hadimani and Malleshi, 1993; Chethan and Malleshi, 2007). Finger millet is important for pregnant and breastfeeding women and children’s nutrition. It plays a significant role in the economies of marginal farmers. Furthermore, finger millet straw is an excellent animal feed, containing up to 60% digestible elements. The seed coat also has anti-cancer and anti-diabetic properties owing to its high polyphenol content.

Nutrients play a significant role in plant growth and development. The scarcity of nutrients in the plants caused an irreversible change. Presently used fertilizers especially nitrogenous fertilizers in crops are less efficient due to their losses in the form of volatilization, surface runoff, leaching and gaseous form. The scarcity of nutrients are more severe when plants are suffering from water deficiency because most of the mineral uptake from the soil to plant cell take the same pathway as a water flow. The application of nanomaterials to crops would revolutionize farming practices by reducing the adverse environmental effects of modern agricultural activities and improving nutrient use efficiency (NUE), grain quality and crop yield (Liu and Lal, 2015; Mahil and Kumar, 2019). Nanotechnology can provide a workable solution to control the difficulties associated with increasing N-based fertilizer usage efficiency (Zulfiqar et al., 2019). It is anticipated to cause a paradigm shift in NUE, resulting in increased agricultural productivity (Ladha et al., 2005). In this context, sequential research was undertaken by synthesizing CaP-U NPs. In comparison to bulk counterparts, CaP-U NPs exhibit greater reactivity and surface area. Considering the above facts, the current study was conducted to reveal the Plant growth promoting potential of urea-doped calcium phosphate nanoparticles (CaP-U NPs) in finger millet (*Eleusine coracana* (L.) Gaertn.) under irrigated and drought stress conditions: an emerging fertilization technique under climate change scenario.

Nanofertilizer such as U-ACP nanoparticles were used as a nitrogen source for *Vitis vinifera* L. (Gaiotti et al., 2021). N-doped ACP NPs with half the absolute N-content than in conventional urea treatment promote the formation of an equivalent amount of root and shoot biomass, without nitrogen depletion (Carmona et al., 2021). The high nitrogen use efficiency (up to 69%) and a cost-effective preparation method support the sustainable real usage of N-doped ACP as a nano fertilizer. In a field experiment, the use of calcium phosphate NPs doped with urea (U-ACP) for the fertilization of *Triticum durum* plants, indicated that yields and quality of the crops treated with the nanoparticles at reduced nitrogen dosages (by 40%) were unaltered in comparison to positive control plants, which were given the minimum N dosages to obtain the highest values of yield and quality in fields. In light of these reports here bring to light the possibility of using engineered nanoparticles to deliver nitrogen to plants more safely and efficiently. However, further research is still needed to secure the most suitable application protocols for real agricultural practices (Ramírez-Rodríguez et al., 2020b). Nano-Urea applied to *Pennisetum glaucum* L. at 30 and 45 DAS, significantly increased plant height, dry matter accumulation, chlorophyll content and nitrogen content (Sharma et al., 2022).

Indeed, drought continues to ravage different regions of the globe, with devastating consequences on soil nutrient bioavailability and crop productivity. Nano NPK improved photosynthetic rate, stomatal conductance, CO_2_ concentration, water use efficiency and relative water content. The chemical composition (plant pigments, total carbohydrates, total phenolic, tannin, total flavonoids, oil constituents, macro and micro-elements) with indigenous hormones (gibberellic acid GA3 and abscisic acid ABA) and antioxidant enzymes (peroxidase and superoxide dismutase) were also positively affected (Mahmoud and Swaefy, 2020). Furthermore, Ca2+ improved maize photosynthesis (45%), stomatal conductance (47%), and accumulation of total soluble sugars (20%) along with the decline in H_2_O_2_ content (23%) (Naeem et al., 2018). hydroxyapatite nanoparticles foliar application in *Adansonia digitata* provide a significant increase in plant growth characteristics (Soliman et al., 2016).

Mechanistically, Research has shown that increased nitrogen (N) improves crop drought tolerance and significant impact on photosynthesis. The nitrogen in plants influenced the water conductivity increasing the accumulation of osmoprotectants and antioxidants (Chang et al., 2016; Zhong et al., 2017; Song et al., 2019). But the majority of supplied N is lost through leaching, volatilization and denitrification leading to a reduction in crop N usage efficiency (Hussain et al., 2019; Pan et al., 2021). The objectives of the present study are to: i) synthesize and characterize urea-doped calcium phosphate nanoparticles (CaP-U NPs); ii) determine whether CaP-U NPs can mitigate the impact of drought stress on the performance of finger millet; and iii) evaluate whether using a lower dose of CaP-U NPs. Collectively, all effects were compared with those of bulk urea to determine the significance of nanoscale size.

## 2 Material and methods

### 2.1 Preparation and characterization of CaP-U NPs

To synthesize CaP-U NPs, urea (70%) was dissolved in a beaker containing distilled water (DW) and kept on a magnetic stirrer until proper mixing. Subsequently, calcium hydroxide [Ca(OH)_2_] was added to the beaker. Afterward, orthophosphoric acid (H_3_PO_4_) was added drop by drop into the beaker containing suspension. Field Emission Scanning Electron Microscope (JEOL FE-SEM) was used to characterize the external morphology of the nanoparticles. The shape and size of the NPs were determined using Transmission Electron Microscopy (TALOS HR-TEM) facility at AIIMS, Delhi. Furthermore, the size and shape of NPs were determined using X-Ray Diffractometer (Bruker). Sonics VCX 750 ultrasonicator (750-watt power and 20kHz frequency) was used to prepare a homogenous solution of CaP-U NPs.

### 2.2 Pot experimental setup

Seeds of finger millet (*var.*VL-379) were procured from Vivekanand Parvartiya Krishi Anusandhan Sansthan (VPKAS), Almora, Uttarakhand, India. For the experiment, finger millet seeds were surface-disinfected by immersion, first in 3 per cent sodium hypochlorite and then in 70 per cent ethanol for 3 and 1 min, respectively. The seeds were then washed thoroughly three times with sterile distilled water. For germination, the seeds were kept on sterilized Petri dishes containing one sheet of sterilized paper moistened with sterilized distilled water and placed in an incubator at 30^°^C for 2 days. All steps were carried out aseptically. Before seed sowing, the pots were filled with sandy loam soil: FYM (3:1). After proper seedling establishment in the pots, 6 seedlings were maintained in each pot till the experiment. CaP-U NPs were tested in the glasshouse to see their ability to increase finger millet growth under irrigated and drought conditions. After seed sowing, in both conditions, 3 treatments were divided into 3 sets; in the first set, pots were untreated, in the second set, pots were treated with foliar spray of bulk urea (0.1 and 0.5%) and in the third set, pots were treated with foliar spray of CaP-U NPs (0.1 and 0.5%) at 15 and 30 days after sowing (DAS). Each treatment was maintained in three replications.

### 2.3 Determination of plant parameters

#### 2.3.1 Plants’ vegetative development parameters

The observation concerning the length of shoot and root, fresh weight of shoot root, dry weight of shoot-root ratio and leaf ratio was recorded at 45 days after sowing. For dry weight, plant samples were kept in an oven at 75°C until constant weight. Shoot and root length was measured from the collar region to the tip of the flag leaf and from the coleoptile region to the tip of the root using a meter scale and expressed in centimeters (cmLeaf area was calculated by measuring the length and width of the leaves per replication. It is multiplied by the total number of small and medium leaves separately. The total leaf area per plant was calculated by the formula given below:

Total leaf area per plant (cm^2^) = Leaf area of small leaf (cm^2^) + Leaf area of medium leaf (cm^2^).

#### 2.3.2 Physiological parameters

##### 2.3.2.1 Estimation of chlorophyll content

Chlorophyll a (Chl a) and chlorophyll b (Chl b) content was estimated by Arnon (1949) method. Fresh leaf of the plant (0.1 g) was collected and placed in a test tube, then added to 10 ml of 80% acetone, sealed with parafilm to prevent evaporation, and kept in the dark for 24 hours. The amounts of Chl a and Chl b were determined by a UV-Visible spectrophotometer at wavelengths 663 nm and 645 nm. The concentrations of chlorophyll a and chlorophyll b (mg g^-1^ FW) in leaf tissues were determined using the following equations:


$$\text{Chl}\;\text{a}=\frac{(12.7\text{XA}663)-(2.69\text{XA}645)\text{XV}}{\text{WX}1000}$$

$$\text{Chl}\;\text{b}=\frac{(22.9\text{XA}645)-(4.68\text{XA}663)\text{XV}}{\text{WX}1000}$$

A = Absorbance at specific wavelength, V = Final volume of chlorophyll extract in 80 percent acetone, W = Fresh weight of tissue extracted (g).

##### 2.3.2.2 Estimation of proline content

Proline content was estimated by Bates et al. (1973) method. First, the plant sample’s fresh leaf (0.2 g) was homogenized in 2.0 ml of 3 percent sulphosalicylic acid (w/v) and centrifuged to remove the residue. After this, 2 ml of leaf extract was treated with 2 ml glacial acetic acid and 2 ml acid ninhydrin for 60 minutes at 100°C. Finally, an ice bath terminated the reaction, and the proline was extracted with 4 ml of toluene. Sample absorbance was measured at 520 nm, and the quantity of proline was calculated using a standard curve. The results were represented in µg free proline per Gram fresh weight (FW).

##### 2.3.2.3 Measurement of malondialdehyde concentration

The MDA content was estimated using Heath and Packer (1968) method. First, the fresh leaf sample (0.3 g) was homogenized in 4 ml of tricholoroacetic acid (0.1 percent). The homogenized sample was centrifuged at 10000 rpm for 15 min. at 4°C, the supernatant was used to estimate MDA. Next, the 0.3 ml of extract was mixed with 1.2 ml of 0.5percent (w/v) 2-thiobarbiturie acid (TBA) prepared in trichloroacetic acid (TAC) (20 percent). The mixture was incubated at 95°C for 30 min. The reaction was terminated by putting the test tubes in an ice bath **quickly** and then cool samples were centrifuged at 10000 rpm for 10 min. The absorbance of the clear supernatant was recorded at 532 nm and 600 nm. Absorbance at 600 nm is subtracted from the absorbance at 532 nm for non-specific absorbance. The MDA concentration was calculated by an extinction coefficient of 155 mM^-1^ cm^-1^.

##### 2.3.2.4 Estimation of hydrogen peroxide

The H_2_O_2_ content was determined by Alexieva et al. (2001) method. Hydrogen peroxide was detected spectrophotometrically after interaction with potassium iodide (KI). Leaf samples (0.1g) were homogenized in 2.0 ml of 0.1 percent trichloroacetic acid (TCA). The reaction mixture included 0.5 ml of supernatant, 0.5 ml of potassium phosphate buffer (0.1 M), and 2 ml of KI solution (1 M). The reaction was carried out in complete darkness for 1 hour and the absorbance was measured at 390 nm. The amount of H_2_O_2_ was determined using a standard curve generated with various dilutions of a working standard of 100 µM of H_2_O_2_.

##### 2.3.2.5 Estimation of total phenol

The total phenol content was estimated by Zieslin and Ben Zaken (1993) method. Fresh leaf sample (0.2 g) were homogenized in 4 ml of 80 percent methanol, heated at 80°C for 20 min, and centrifugated at 10,000 rpm. Next, 1 mL of methanolic extract containing phenol was mixed with 5 mL of distilled water and 250 µl of Folin-Ciocalteau reagent (1 N) in a 5 mL vial. Finally, 1 mL saturated sodium carbonate (20 percent) was added immediately, and the mixture was incubated at 25°C for 30 min. A Genesys 10S UV–Vis Spectrophotometer was used to measure the absorbance of the generated blue color at 725 nm. Phenolic content is represented as µg GAE g^-1^ fresh weight.

### 2.4 Estimation of antioxidant enzymes of plants

#### 2.4.1 Preparation of enzyme extracts

For determination of antioxidant enzyme activities, 0.5 g fresh leaf sample was homogenized with a pestle in an ice-cold mortar in 5 ml cold buffer containing: 50 mM potassium phosphate buffer (pH 7.0), 2 mM ethylene diamine tetra acetic acid (EDTA) and 1% polyvinyl-pyrrolidone (PVP). The whole extraction procedure was carried out at 4°C. The homogenate was centrifuged at 10,000 rpm for 30 min at 4°C and the supernatant collected was used to assay enzyme activity.

#### 2.4.2 Estimation of superoxide dismutase activity

SOD activity was assay based on the ability of superoxide dismutase to inhibit the reduction of nitro-blue tetrazolium (NBT) (Beauchamp and Fridovich, 1971). The reaction mixture (3 ml) for the SOD assay contained 50 mM Na-phosphate buffer (pH 7.8), 13 mM L-methionine, 75 uM NBT, 10 µM EDTA, 2.0 µM riboflavin, and 0.1 ml enzyme extract. The reaction mixture was incubated in test tubes for 10 minutes at 35°C in 4000 lux. After illumination, the tubes were covered with black cloth, and absorbance was measured at 560 nm. The activity is represented as a unit per mg protein (unit mg^-1^ protein).

#### 2.4.3 Estimation of guaiacol peroxidase activity

Peroxidase activity was determined by a pyrogallol method developed by Kar and Mishra (1975). H_2_O_2_ oxidized a colorless pyrogallol compound into a colored purpurogallin compound. 100 mM potassium phosphate buffer (pH 7.2), 0.1 mM EDTA, 5 mM guaiacol, 15 mM H_2_O_2_, and 100 µl enzyme extract were used in the reaction. At 470 nm, an increase in absorbance was recorded every 10 seconds. The amount of enzyme activity is determined by the formation of tetra-guaiacol. The activity is represented as µmol tetra-guaiacol formed per min. per mg protein.

#### 2.4.4 Estimation of ascorbate peroxidase activity

Ascorbate peroxidase (APX) activity was determined by Nakano and Asada (1981) method. The enzyme was extracted in 50 mM phosphate buffer for APX activity. The APX reaction mixture contained 50 mM phosphate buffer, 0.5 mM ascorbic acid, 0.2 mM EDTA, and enzyme extract. The reaction began after the addition of 0.1 mM H_2_O_2_. Absorbance was measured spectrophotometrically at 290 nm and the reduction in absorbance was recorded for up to 90 seconds after the reaction began. Therefore, the activity is represented as nmol per minute per mg protein.

## 3 Statistical analysis

Data and results were represented in means, which were statistically examined by SPSS (Statistical package for the social science) software comparing variance (ANOVA) function. Duncan’s multiple range test was used to compare the treatment mean values at the P ≤ 0.05 significant level. Principal component analysis (PCA) and Pearsion correlation were done using Origin and R-square (version 4.1.2) to demonstrate the correlation between the various plant growth parameters and defense enzyme and their relationship with the different treatments.

## 4 Results

### 4.1 Synthesis and characterization of CaP-U NPs by FE-SEM, TEM and XRD analysis

**Figure 1:**
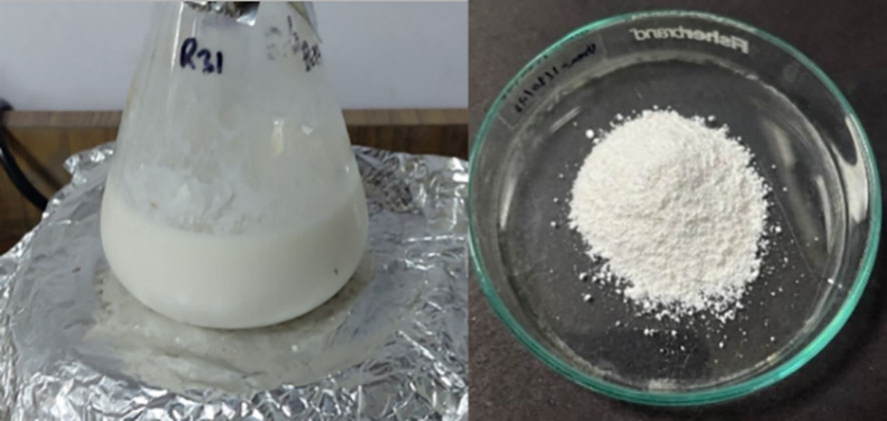
Visual examination of generated CaP-U NPs.

**Figure 2:**
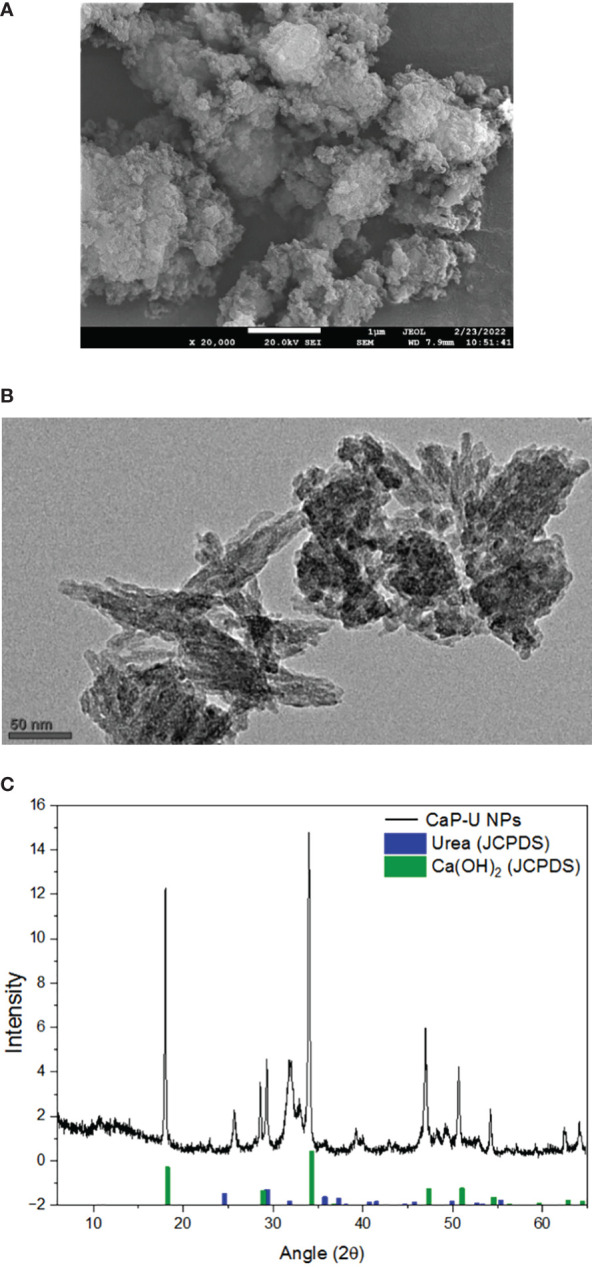
**(A)** CaP-U NPs under FE-SEM microscope **(B)** TEM-image showing CaP-U NPs with irregular morphology (scale bar 50 nm) (Tiny rods with diameters in the range from 15 to 50 nm and lengths ranging from 100 to 200 nm) **(C)** XRD analysis of CaP-U NPs.

In the visual examination of the CaP-U NPs urea solution, the color changed from transparent to white, indicating the formation of CaP-U NPs (**Figure 1**). The morphology of CaP-U NPs was determined by FE-SEM (magnification 20000 x) (**Figure 2A**). There are no areas of phase separation in urea and calcium phosphate, indicating the successful formation of an encapsulated nanocomposite between the two nano matrices. HR-TEM further confirmed this formation. TEM analysis depicted rod-like, irregularly shaped smaller particles of CaP-U NPs with diameters in the range from 15 to 50 nm and lengths ranging from 100 to 200 nm (**Figure 2B**). According to TEM analysis, the rods are covered with urea at the nanoscale. High-resolution images showed the partial porous structure of CaP-NPs, which was then executed to load urea onto it. X-ray diffraction (XRD) patterns of the powder samples were recorded using Cu K_α_ radiation (= 1.54178 Å). Spectra were recorded in the 2θ range from 10° to 60° with a step size (2θ) of 0.02 and a counting time of 0.5 s. The graph with the sharpest peaks, like the (101) plane, corresponds to the pure hexagonal structure of Ca(OH)_2_ (JCPDS No. 84-1276), as indicated by the green bar diagram. The graph displays the diffraction peaks of urea, with the maximum intensity peak (111) corresponding to a pure tetragonal structure that matches JCPDS No. 99-101-0067 and is displayed as a blue bar diagram. Further evidence that there isn’t any characteristic peak of the crystalline impurity is provided by the crystal phases of the mixture of Ca(OH)_2_ and urea displayed in the graph (**Figure 2C**).

### 4.2 Pot experiment

In the pot experiment, the observation concerning plant growth parameters and biochemical analysis was recorded at 45 days after sowing (DAS) under both irrigated and drought conditions.

#### 4.2.1 Shoot length

**Figure 3:**
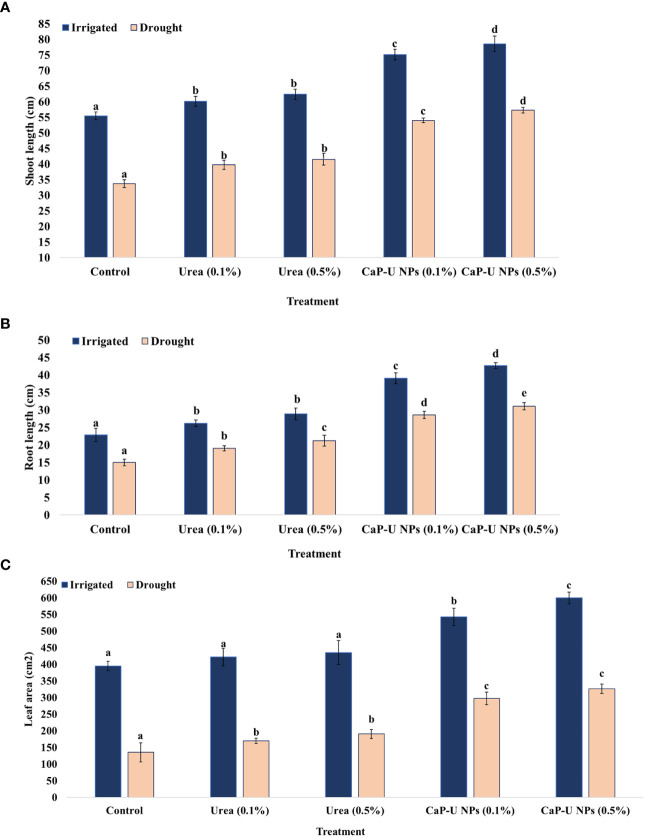
Effect of different concentrations of CaP-U NPs on **(A)** shoot length, **(B)** root length, and **(C)** leaf area of *Eleusine coracana* under irrigated and drought conditions. Results are indicated as means of three replications and vertical bars express the standard deviation (SD) of the means. Different letters denote significant differences among treatment outcomes taken at the same time interval according to Duncan’s multiple range test at P≤ 0.05.

In this experiment, at 45 DAS, the maximum shoot length was recorded at 0.5% conc.of CaP-U NPs, with 29.4 and 41% increase followed by 0.1% conc. of CaP-U NPs, with 26.2 and 37.5% increase under irrigated and drought conditions respectively. In contrast, in case of urea, the maximum shoot length was recorded at 0.5% conc., with 11.1 and 18.7% increase followed by urea 0.1% conc., with 7.8 and 15% increase compared to control under irrigated and drought conditions respectively (**Figure 3A**).

#### 4.2.2 Root length

Water deficiency first affects the roots (Hsiao and Xu, 2000); deep-rooted plants are better adapted to drought conditions (Ho et al., 2005). In this experiment, at 45 DAS, the maximum root length was recorded at 0.5% conc. of, with 46.4 and 51% increase followed by 0.1% conc. of CaP-U NPs treatment, with 41.4 and 47.4% increase. In case of urea, the maximum root length was recorded at 0.5% conc., with 20.7 and 29.3% increase followed by 0.1% conc. urea, with 12.6 and 21% increase compared to control, under irrigated and drought conditions respectively (**Figure 3B**).

#### 4.2.3 Leaf area

Under drought conditions, the leaf area of the plants was significantly impacted. Prolonged drought stress caused a significant reduction in the leaf area due to decreased cell division and cell expansion (Koch et al., 2019). In this experiment, at 45 DAS, in the case of CaP-U NPs treatment, the maximum leaf area was recorded at 0.5% conc., with 34.1 and 58.6% increase followed by 0.1% conc. of CaP-U NPs, with 27.2 and 54.5% increase under irrigated and drought conditions respectively, compared to control. In the case of urea, the maximum leaf area was recorded at 0.5% conc., with 9.3 and 29.1% increase followed by 0.1% conc.of urea, with 6.4 and 20.2% increase compared to control, under irrigated and drought conditions respectively (**Figure 3C**).

#### 4.2.4 Shoot fresh weight

**Figure 4:**
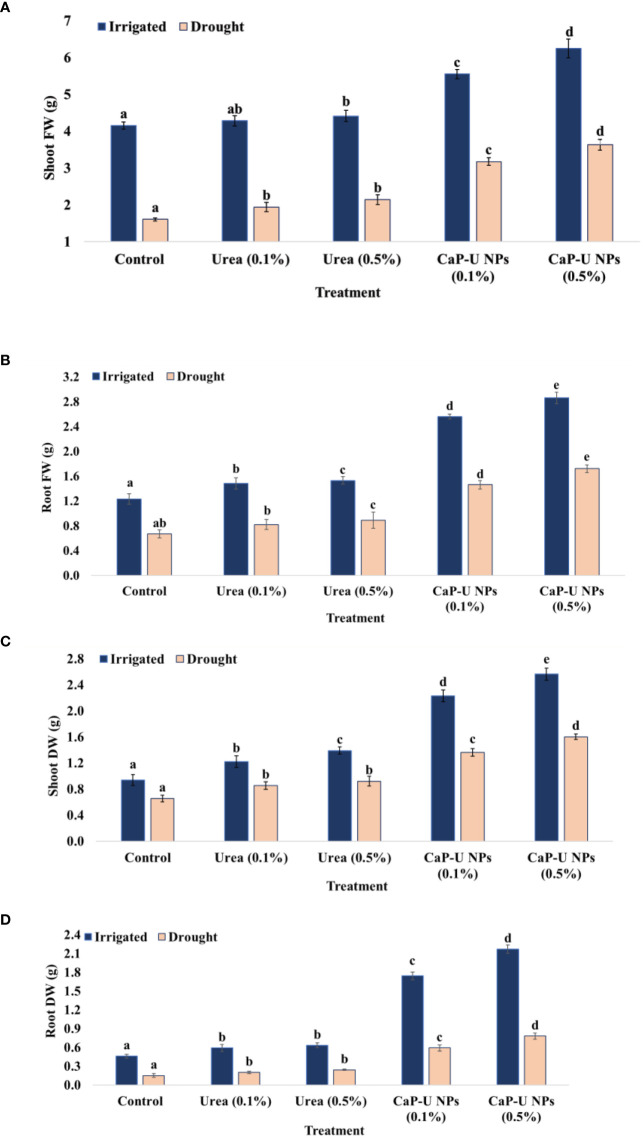
Effect of different concentrations of CaP-U NPs on **(A)** shoot fresh weight, **(B)** root fresh weight, **(C)** shoot dry weight and **(D)** root dry weight of *Eleusine coracana* under irrigated and drought conditions. Results are indicated as means of three replications and vertical bars express the standard deviation (SD) of the means. Different letters denote significant differences among treatment outcomes taken at the same time interval according to Duncan’s multiple range test at P≤ 0.05.

Plants with lesser biomass reduction under drought stress are drought-tolerant (Passioura, 2002). In this experiment, at 45 DAS, in the case of foliar spray of CaP-U NPs, the maximum shoot fresh weight was recorded at 0.5% conc., with 33.6 and 55.8% increase followed by 0.1% conc. of CaP-U NPs with 25.3 and 49.7% increase under irrigated and drought conditions respectively. While in the case of urea the maximum shoot fresh weight was recorded at 0.5% conc., with 6 and 25.3% increase followed by 0.1% conc. of urea, with 3 and 17.3% increase compared to control, under irrigated and drought conditions respectively (**Figure 4A**).

#### 4.2.5 Shoot dry weight

Dry weight loss in drought conditions has been more associated with shoot than root (Mohammadian et al., 2005). In this experiment, at 45 DAS, in CaP-U NPs treatment, the maximum shoot dry weight was recorded at 0.5% conc., with 63 and 59.1%i increase followed by 0.1% conc. of CaP-U NPs, with 58 and 51.8%i increase; while in the case of urea, the maximum shoot dry weight was recorded at 0.5% conc., with 32.6 and 28.8% increase followed by treatment with 0.1% conc. urea, with 23 and 23.3% increase compared to control under irrigated and drought conditions respectively (**Figure 4C**).

#### 4.2.6 Root fresh weight

Drought stress has been reported to reduce roots’ fresh and dry weight (Mohammadkhani and Heidari, 2008). In this experiment, at 45 DAS, in case of CaP-U NPs treatment, the maximum root fresh weight was recorded at 0.5% conc., with 57 and 61% increase followed by 0.1% conc. of CaP-U NPs, with 52 and 54.1% increase; while in urea treatment, the maximum root fresh weight was recorded at 0.5% conc., with 19.6 and 24.6% increase followed by 0.1% conc. of urea, with 16.9 and 18.2% increase compared to control, under irrigated and drought conditions respectively (**Figure 4B**).

#### 4.2.7 Root dry weight

In the case of CaP-U NPs, at 45 DAS, the maximum root dry weight was recorded at 0.5% conc., with 78 and 80.7% increase followed by 0.1% conc. of CaP-U NPs, with 73 and 74.6% increase; while in the case of urea, the maximum root dry weight was recorded at 0.5% conc. with 27 and 37.5% increase followed by 0.1% conc. of urea, with 22 and 25% increase compared to control, under irrigated and drought conditions respectively (**Figure 4D**).

#### 4.2.8 Chlorophyll content

Under drought conditions, producing excessive reactive oxygen species might cause a decreased chlorophyll content. Therefore, retaining green leaves under drought conditions is considered an important parameter for drought tolerance (Deblonde and Ledent, 2001).

**Figure 5:**
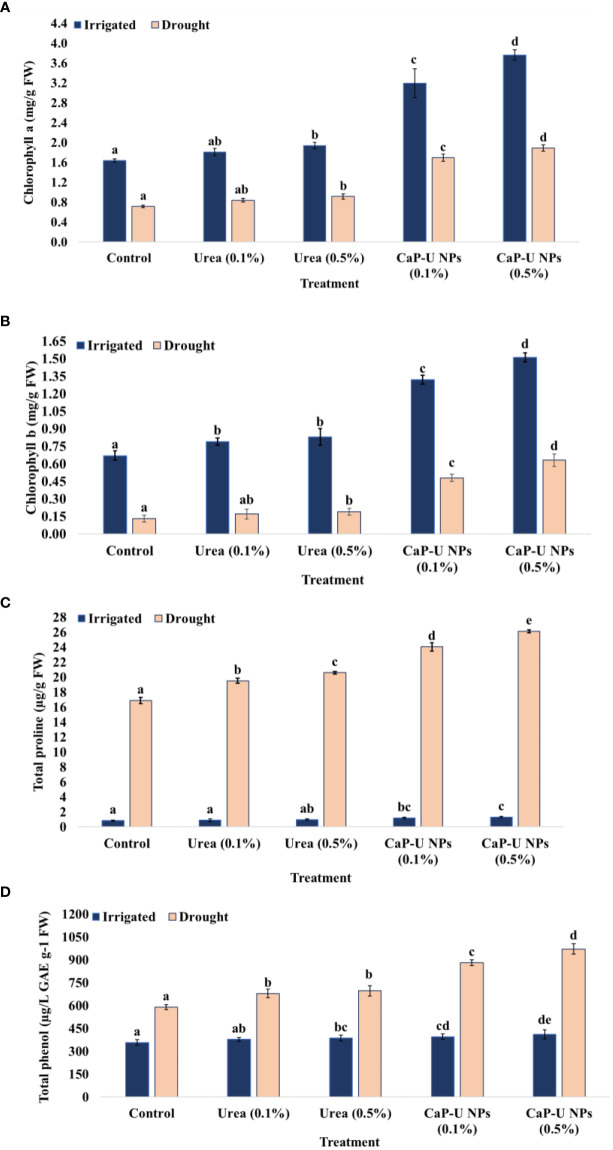
Effect of different concentrations of CaP-U NPs on **(A)** chlorophyll a, **(B)** chlorophyll b, **(C)** total proline content, and **(D)** total phenol content of *Eleusine coracana* under irrigated and drought conditions. Results are indicated as means of three replication and vertical bars express the standard deviation (SD) of the means. Different letters denote significant differences among treatment outcomes taken at the same time interval according to Duncan’s multiple range test at P≤ 0.05.

At 45 DAS, the maximum Chl a was recorded at 0.5% conc. of CaP-U NPs, with 56.4 and 62.3% increase followed by 0.1% conc of CaP-U NPs, with 48.6 and 58% increase. In contrast, with application of urea, the maximum Chl a was recorded at 0.5% conc., with 15.5 and 21.9% increase followed by 0.1% conc. urea, with a 9.4 and 15.1% increase compared to control, under irrigated and drought conditions respectively. Similarly, maximum Chl b was recorded at 0.5% conc. of CaP-U NPs, with 55.6 and 79.4% increase followed by 0.1% conc. resulting in a 49.2 and 72.9% increase. In the case of urea, the maximum Chl b was recorded at 0.5% conc., with 19.3 and 31.6% increase followed by treatment with 0.1% conc. of urea resulting in a 15.2 and 23.5% increase compared to control, under irrigated and drought conditions, respectively (**Figures 5A, B**).

#### 4.2.9 Total proline content

During drought stress, plants accumulate more proline than in normal (no drought) conditions (Shakeel et al., 2011). In drought stress, a high level of leaf proline plays a crucial role in maintaining the osmotic potential of the tissues, which prevents severe dehydration. At 45 DAS, in the case of CaP-U NPs, the maximum total proline content was recorded at 0.5% conc., with a 35.4% increase followed by 0.1% conc. of CaP-U NPs, with a 29.8% increase; while in the case of urea, the maximum total proline content was recorded at 0.5% conc., with a 17.9% increase followed by 0.1% conc. of urea, with a 13.4% increase compared to control under drought conditions. There were no significant differences between control and treated plants under irrigated conditions (**Figure 5C**).

#### 4.2.10 Total phenol content

Drought induces oxidative stress in plants, which results in ROS production. Phenols and Flavonoids are examples of adaptive natural compounds that enable plants to scavenge ROS (Yadav et al., 2021). Increased synthesis of phenols promotes drought tolerance in plants (Verma and Deepti, 2016). n this experiment, at 45 DAS, in the case of CaP-U NPs, the maximum total phenol content was recorded at 0.5% conc., with a 39.3% increase followed by CaP-U NPs at 0.1% conc., with a 33.1% increase. In case of urea, the maximum total phenol content was recorded at 0.5% conc., with a 15.3% increase followed by urea at 0.1% conc., with a 13.2% increase compared to control, under drought conditions. Thus, in the present study, TPC levels did not differ significantly under irrigated conditions, whereas TPC levels increased under drought-stressed conditions (**Figure 5D**).

### 4.3 Enzymatic antioxidant analysis

**Figure 6:**
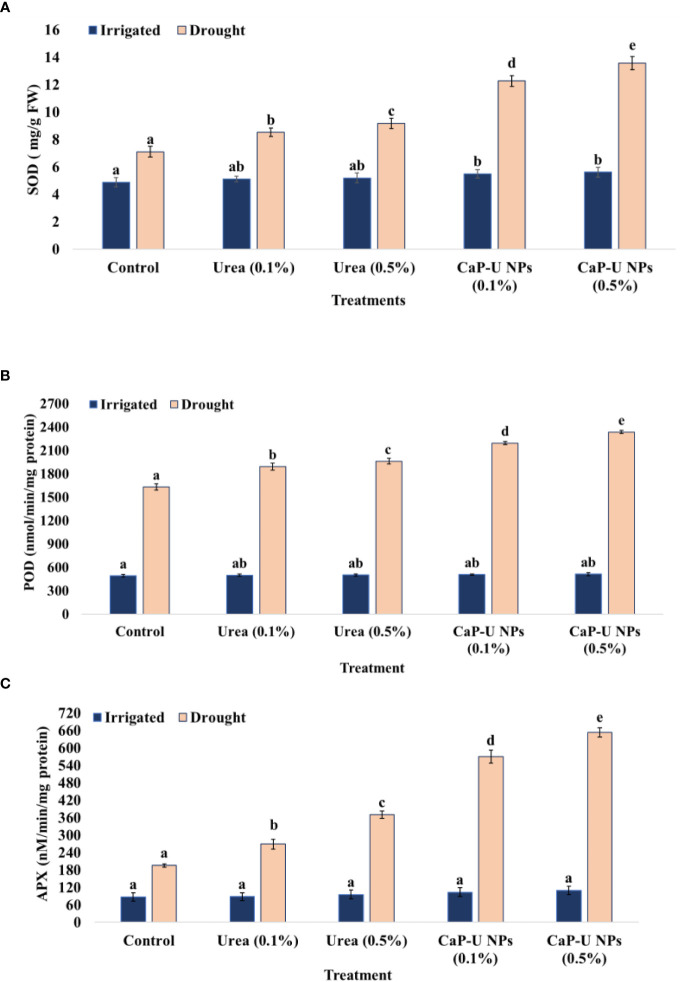
Effect of different concentrations of CaP-U NPs on **(A)** superoxide dismutase, **(B)** peroxidase (POD), and **(C)** ascorbate peroxidase (APX), of *Eleusinecoracana* (L.) Gaertn under irrigated and drought conditions. Results are indicated as means of three replication and vertical bars express the standard deviation (SD) of the means. Different letters denote significant differences among treatment outcomes taken at the same time interval according to Duncan’s multiple range test at P≤ 0.05.

Under irrigated conditions, antioxidant enzyme activity did not differ significantly between the control and treatments. However, the activity of SOD, POD and APX was significantly increased in finger millet plants treated with foliar spray in response to drought stress compared to untreated control (**Figures 6A–C**). Correlation coefficient analysis of genes/proteins with NPs further revealed that the higher binding potential of the NPs on the proteins/genes would result in more transcriptional modulation and more expression of genes (Kumar et al., 2015; Chandra et al., 2021).

#### 4.3.1 Super oxide dismutase

Abiotic stresses have been associated with a progressive rise in the activity of SOD, APX and CAT (Caverzan et al., 2016). Increased antioxidant enzymes showed plant adaptation to counteract increased oxidant production (Bagheri et al., 2015). SOD is the primary scavenger of superoxide and plays a vital role in defense against cellular damage caused by environmental stress (Ren et al., 2016). SOD levels did not differ significantly under irrigated conditions, whereas SOD levels increased under drought-stressed conditions. In this experiment, at 45 DAS, the maximum SOD was recorded at 0.5% conc. of CaP-U NPs, with a 47.7% increase followed by 0.1% conc. of CaP-U NPs, with a 42.1% increase; while with urea treatment, maximum SOD was recorded at 0.5% conc., with a 22.6% increase followed by 0.1% conc. of urea, with a 16% increase compared to control under drought conditions (**Figure 6A**).

#### 4.3.2 Peroxidase

POD levels did not differ significantly under irrigated conditions, whereas POD levels increased under drought conditions. In this experiment, at 45 DAS, in the case of CaP-U NPs treatment, the maximum POD activity was recorded at 0.5% conc., with a 30.2% increase followed by 0.1% conc. of CaP-U NPs, with a 25.7% increase, while in the case of urea, maximum POD was recorded at 0.5% conc., with a 16.9% increase followed by 0.1% conc. of urea, with a 13.9% increase compared to control, under drought conditions (**Figure 6B**).

#### 4.3.3 Ascorbate peroxidase

APX levels did not differ significantly under irrigated conditions, whereas APX levels increased under drought conditions. In this experiment, at 45 DAS, in the case of CaP-U NPs, the maximum APX was recorded at 0.5% conc., with a 70% increase followed by 0.1% conc. of CaP-U NPs, with a 65.6% increase, while with urea treatment, the maximum APX was recorded at 0.5% conc., with a 47.1% increase followed by 0.1% conc. urea, with a 27.1% increase compared to control, under drought conditions (**Figure 6C**).

#### 4.3.4 Malondialdehyde content

**Figure 7:**
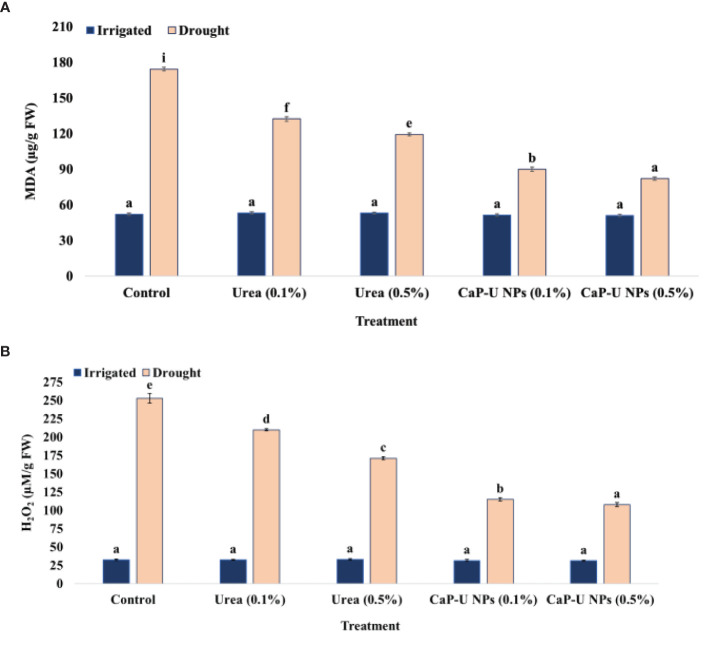
Effect of different concentrations of CaP-U NPs on **(A)** Malondialdehyde (MDA), and **(B)** Hydrogen peroxide (H_2_O_2_) of *Eleusinecoracana* (L.) Gaertn under irrigated and drought conditions. Results are indicated as means of three replication and vertical bars express the standard deviation (SD) of the means. Different letters denote significant differences among treatment outcomes taken at the same time interval according to Duncan’s multiple range test at P≤ 0.05.

MDA levels did not differ significantly under irrigated conditions, whereas MDA levels increased under drought stress conditions. In this experiment, at 45 DAS, in the case of N-CaP U NPs, the minimum MDA was recorded at 0.5% conc., with a 112.5% decrease followed by 0.1% conc. of CaP-U NPs, with a 93.7% decrease, while in the case of urea, the minimum MDA was recorded at 0.5% conc., with a 46.1% decrease followed by 0.1% conc. of urea, with a 31.8% decrease compared to control, under drought conditions (**Figure 7A**).

#### 4.3.5 Hydrogen peroxide

Dismutation of superoxide radicals results in hydrogen peroxide accumulation under stress conditionsH_2_O_2_ levels did not differ significantly under irrigated conditions, whereas H_2_O_2_ levels increased under drought stress conditions. In this experiment, at 45 DAS, in the case of CaP-U NPs, the minimum H_2_O_2_ was recorded at 0.5% conc. with a 134.5% decrease followed by 0.1% conc. of CaP-U NPs, with a 119.9% decrease; while in the case of urea, the minimum H_2_O_2_ was recorded at 0.5% conc., with a 47.7% decrease followed by 0.1% conc. of urea, with a 20.4% decrease compared to control under drought conditions (**Figure 7B**).

### 4.4 Principal component analysis of plant growth and biochemical data under NP and urea treatment

**Figure 8:**
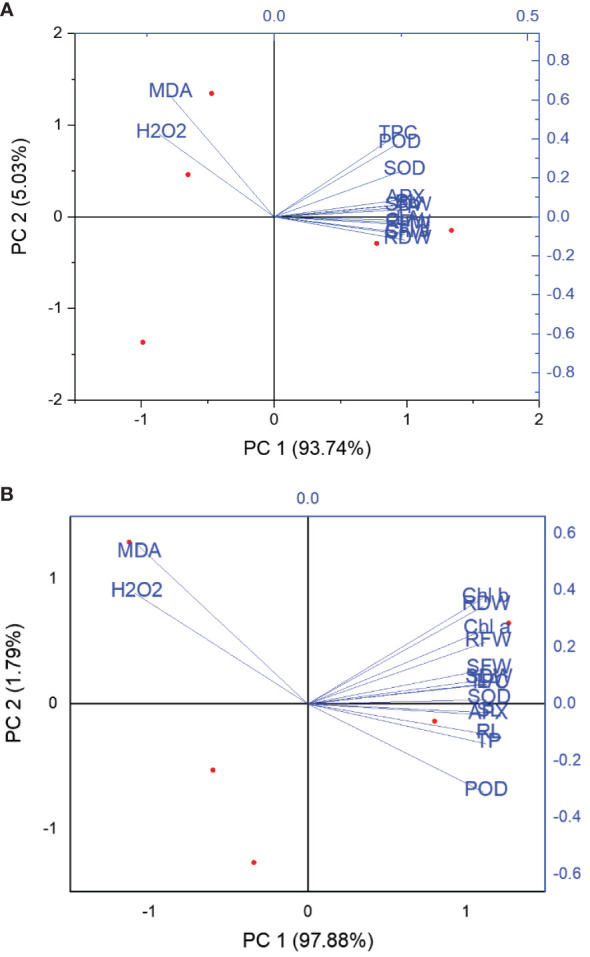
Principal component analysis of plant growth parameters upon application of CaP-U NPsa and urea under **(A)** Irrigated and **(B)** Drought conditions.

PCA analysis was done to establish a relationship between plant growth and biochemical parameters in relation to CaPU NPs and urea applications under both irrigated and drought conditions. The distribution of growth and biochemical parameters in space defined by the first and second PCA dimensions is shown in **Figure 8B**. The PCA comprising two principal components (PC1 and PC2) explained 98.77 and 99.67% of the total variation in irrigated and drought conditions, respectively. Under irrigated conditions, PC1 explained 93.74% and PC2 explained 5.03% of the total variation while in drought conditions PC1, explained 97.88% and PC2 explained 1.79% of the total variation (**Figure 8A**). A strong correlation was observed between various growth and biochemical attributes. Superimposition of five treatments combinations on drought suggested that CaP-U NPs at 0.5 followed by 0.1% provided the highest growth indices and defense-related enzymes, which were significantly different. Further, in the control group, all the growth parameters and enzymatic activity were the least and did not show any correlation with growth and biochemical attributes. Under the irrigated conditions, the normal trend was observed, in which CaP-U NPs at 0.5 followed by 0.1% provided the highest growth indices and defense-related enzymes.

### 4.5 Pearson correlation explains the strength of the growth and biochemical attributes under irrigated and drought conditions influenced by the NP and urea treatment

**Figure 9:**
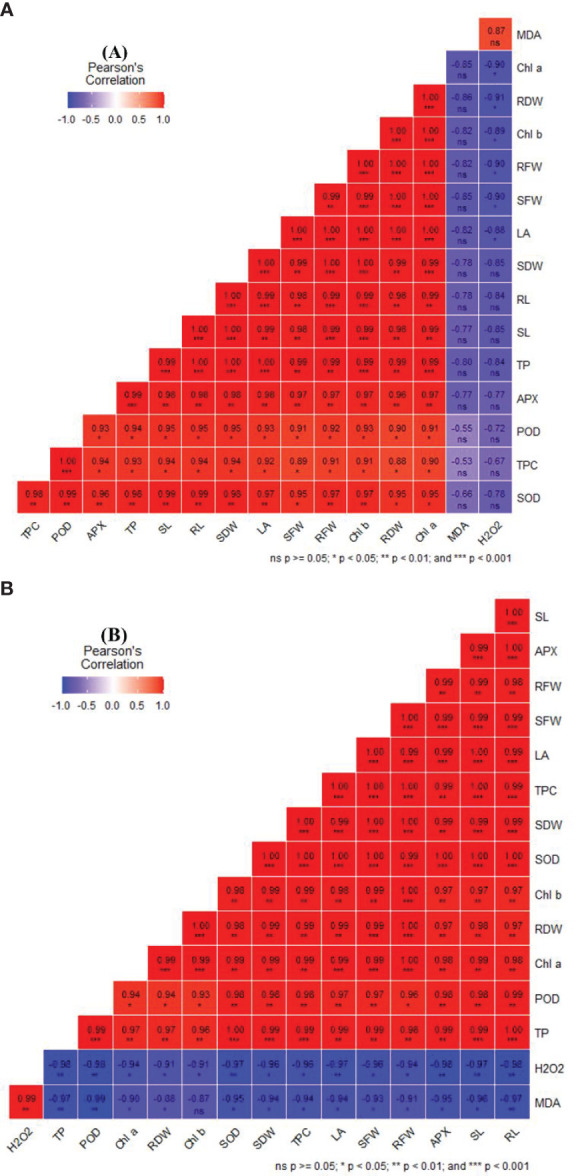
Correlation of different growth and defense parameters upon CaP-U NPs and urea application in finger millet under Irrigated **(A)** and drought **(B)** conditions.

The leaf area of a plant is considered one of the important components of the growth attributes due to its excellent role in photosynthesis accumulation. High leaf areas intercept more light compared to low ones resulting in more food accumulation in the plants which directly influenced plant growth and enzymatic regulations. Under irrigated conditions, leaf area had a significant strong positive correlation with the shoot (0.99) and root (0.99) length, shoot (1.0) and root (1.0) fresh weight, shoot (1.0) and root (1.0) dry weight and also a strong positive correlation with defense enzymes such as Chl a (1.0), Chl b (1.0), SOD (0.97), TPC (0.92), total proline (1.0), APX (0.98) and POD (0.93) while negatively correlated with H_2_O_2_ (-0.88) and MDA (-0.82). However, other growth parameters such as root and shoot length also showed a significant strong positive correlation with leaf area, root fresh weight (0.99 & 0.99), shoot fresh weight, root dry weight (0.98 & 0.98), shoot dry weight (1.0 & 1.0), respectively. It also showed a strong positive correlation with enzymes such as Chl a & b, SOD (0.99 & 0.99), Total proline (1.0 & 0.99), TPC (0.94 & 0.94), APX (0.98 & 0.98) and POD (0.95 & 0.95) but negatively correlated with H_2_O_2_ (-0.84 & -0.85) and MDA (-0.78 & -0.77), respectively (**Figure 9A**).

Under drought conditions, leaf area has a significant strong positive correlation with the shoot (1.0) and root (0.99) length, shoot (1.0) and root (0.99) fresh weight, shoot (0.99) and root (0.99) dry weight as well as showed a strong positive correlation with biochemical parameters such as Chl a (0.99), Chl b (0.98) and defense enzymes such as SOD (1.0), TPC (1.0), total proline (0.99), APX (0.99) and POD (0.97) while negatively correlated with H_2_O_2_ (-0.97) and MDA (-0.94). However, other growth parameters such as root and shoot length also showed a significant strong positive correlation with leaf area (0.99 & 1.0), root fresh weight (0.98 & 0.99), shoot fresh weight (0.99 & 0.99), root dry weight (0.97 & 0.98), shoot dry weight (0.99 & 0.99), respectively. It also showed a strong positive correlation with enzymes such as Chl a (0.98 & 0.99) & Chl b (0.97 & 0.97), SOD (1.0 & 1.0), Total proline (1.0 & 0.99), TPC (0.99 & 1.0), APX (1.0 & 0.99) and POD (0.99 & 0.98) but negatively correlated with H_2_O_2_ (-0.98 & -0.97) and MDA (-0.97 & -0.96), respectively (**Figure 9B**).

## 5 Discussion

**Figure 10:**
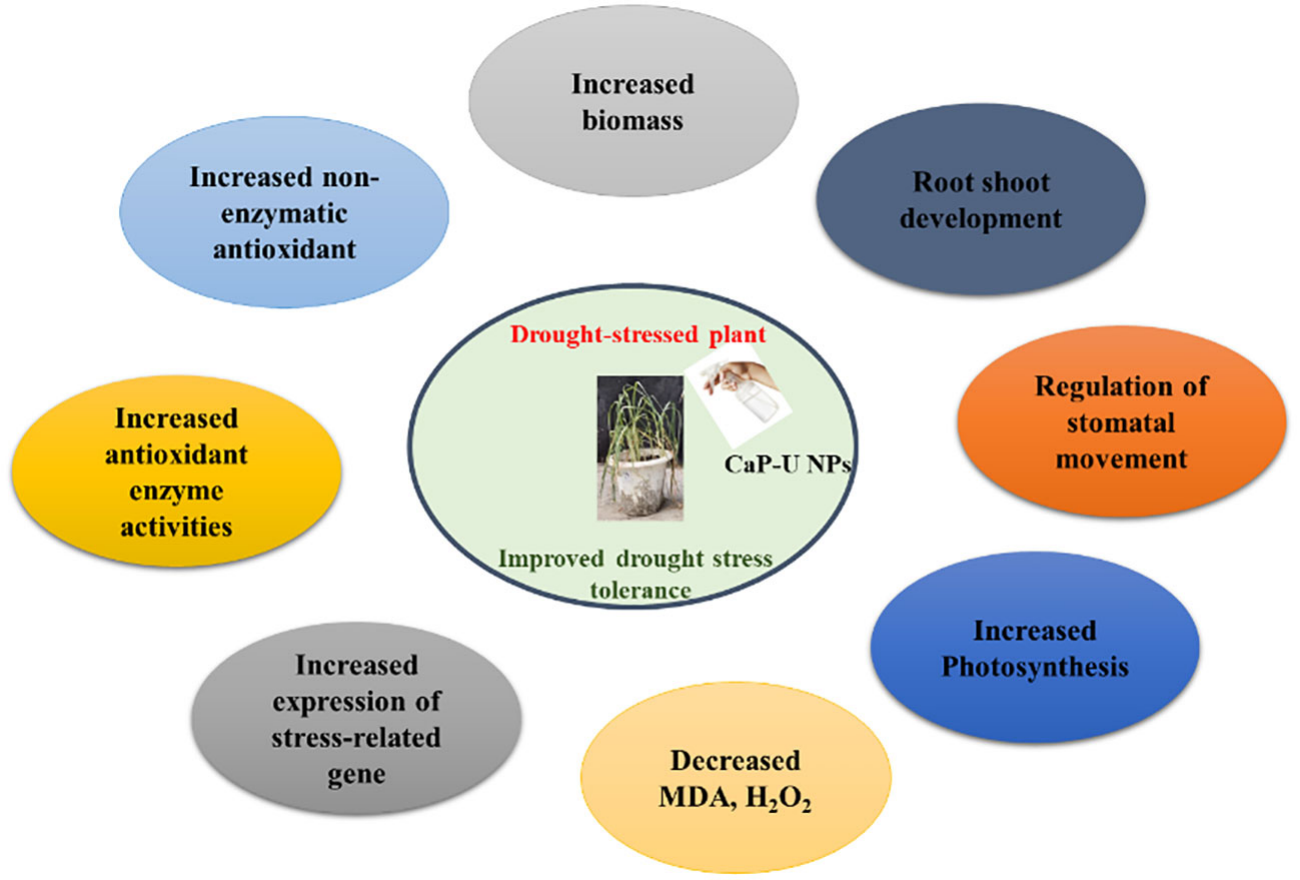
General mechanism of CaP-U NPs induced drought stress tolerance in plants.

Water scarcity significantly influences plant performance by reducing growth, development and other physiological processes by higher accumulation of ROS levels, which causes cell dysfunction in the plants. However, in the present study, applying CaP-U NPs increased the growth, photosynthetic pigments as well as antioxidant enzyme activity by reducing the ROS level under drought-stress conditions. N accumulation in the foliar parts of the plants has a positive correlation with root water conductivity (**Figure 10**). The nitrogen in plants influenced the water conductivity, which is regulated by the expression of the aquaporin gene mainly nodulin 26-like protein (NIPs) and tonoplast intrinsic proteins (TIPs). However, over-expression of the aquaporin genes could enhance plant drought tolerance (Ren et al., 2015; Li et al., 2016). Generally, NIPs were observed to facilitate the transport of water and the efflux of N, as well as the entry of N into cells *via* the plasma membrane, followed by vacuolar loading through TIPs. Vacuolar loading is beneficial for the storage of excess N, and vacuolar unloading can remobilize the N under N starvation conditions (Ding et al., 2018).

Nano-fertilizers application significantly improved uptake through pores or uptake could be facilitated by complexation with molecular transporters, through the creation of new pores, or by exploitation of endocytosis or ion channels (Rico et al., 2011). Nano-fertilizers are nutrient carriers capable of holding bountiful nutrients due to their high surface area and releasing it slowly (Abdel-Aziz et al., 2016). Nano fertilizers control the release of nutrients from the fertilizer granules to improve nutrient utilization efficiency (NUE) while preventing the nutrient ions from getting fixed or lost in the environment (Subramanian et al., 2015; Chhipa, 2017). Nanoscale fertilizers could lead to the more effective delivery of nutrients as their small size may allow them access to various plant surfaces and transport channels. Leaf surfaces are nano- and microstructured surfaces containing cuticular pores and stomata. A study on the penetration of two different sizes particles (43 nm or 1.1 μm diameter) into leaves of *Vicia faba* L. indicated that the nano-sized particles could penetrate the leaf interior through the stomatal pores (Eichert et al., 2008). The stomatous leaf surfaces of *V. faba* and *Prunus cerasus* had an average pore radius ranging from 25 to 100 nm. Once within the plant, cell-to-cell transport within a plant could be facilitated by the plasmodesmata (Zambryski, 2004). Plasmodesmata are nanoscale channels, 50-60 nm in diameter, enabling cell-to-cell communication and transport (Gunning and Steer, 1996). Chitosan-NPK (10%) fertilizer application gives a significant increase in crop index and harvest index as compared to conventional fertilizer used in wheat crops (Abdel-Aziz et al., 2016). Although in *Abelmoschus esculentus*, the application of commercial fertilizer needed more quantity (5 g/week) compared to manufactured nano urea (50 mg/week) (Tarafder et al., 2020). Zeolite Based Nitrogen Nano-fertilizers increase yield and nitrogen content in Maize plants (Manikandan and Subramanian, 2016). Urea nano fertilizers can reduce a minimum of 25% of the recommended dose of conventional urea fertilizer, which may be introduced as a more sustainable and economical agricultural practice (Singh et al., 2023). A study was conducted in the Haryana state of India with a total area of 1225 acres and found that an average yield was recorded 5.35% higher in wheat, 24.24% in sesame and 8.4% in mustard by applying nano fertilizers of nitrogen and zinc along with the organic farming practice (Kumar et al., 2022). If N nano fertilizer was applied at the rate of 60 kg ha^−1^ on Sunflowers would produce the highest seed yield (17.6%) and oil yield (28.7%) as compared to conventional N fertilizer (Handayati and Sihombing, 2019). Half doze of HA-N nano fertilizer shows a similar effect of urea in rice (Bhavani et al., 2020).

CaP-U NPs are primarily used as a regulator rather than a nutrient source because they have the ability to control the release of urea and other nutrients. This controlled release feature allows for more efficient use of the nutrients by the plants. Furthermore, the calcium phosphate component of CaP-U NPs can also increase the availability of essential nutrients such as phosphorus and calcium. This can enhance plant growth and improve crop yields. In the present study, the formation of the CaP-U NPs was confirmed by the color change of the solution. FE-SEM determined the morphology of CaP-U NPs. In FE-SEM Neither urea nor calcium phosphate exhibit any areas of phase separation, indicating that the nanocomposite is successfully encapsulated between the two nano matrices. According to FE-SEM analysis, nano urea particles were of different sizes and possessed a fiberlike structure. These findings are similar to Tarafder et al. (2020) and Kottegoda et al. (2017). According to TEM analysis, the rods are covered with urea at the nanoscale. Carmona et al. (2021) found the existence of irregularly shaped Nano-U-ACP in TEM imaging. Nano NPK TEM analysis confirmed the precipitation of amorphous round-shaped nanoparticles with sizes in the 10-25 nm range (Ramírez-Rodríguez et al., 2020a). The shape of luminous europium-calcium phosphate nanoparticles was round, with sizes far below 100 nm under TEM analysis (Ortiz-Gómez et al., 2020). CaP NPs had a diameter of 26 nm as measured by TEM photomicrographs (Morgan et al., 2008).

Rajonee et al. (2016) designed a nitrogen delivery system wherein hexadecyl trimethyl ammonium bromide (HDTMABr) was used as a surfactant for modifying zeolite, which was then loaded with nitrogen. A pot experiment with *Ipomoea aquatica* was carried out to evaluate the efficiency of this synthesized nanocomposite and the results showed that nitrogen uptake by the plants treated with nanocomposite was higher than those treated with conventional urea. CaP-Urea NPs have a larger specific surface area and reactivity than their crystalline counterparts (bulk) material. In lettuce, foliar spray of the urea and dry yeast extract significantly improved vegetative growth characteristics, pigment content, chemical content as well as head yield compared to untreated control (Abd El Galil et al., 2021).

In the present study, urea is loaded onto the CaP surface and hence released more slowly than highly soluble traditional fertilizers; this helped in plants’ gradual nitrogen absorption. CaP also shows high solubility in neutral or slightly acidic environments and hence can transfer more of these essential ions to the plant, allowing them to be used as multi-nutrient nano fertilizer (Giroto et al., 2017; Kopittke et al., 2019). Calcium plays a significant role in the development of the structural part and in the biochemical process, i.e., signaling of the plants (Sharma et al., 2017). Calcium also gives resistance to plants against fungal infection since it plays a crucial role in stabilizing and strengthening the cell wall (Cesco et al., 2020). In soybean (*Glycine max*), the application of the hydroxyapatite nanoparticles as a rich source of phosphorus was reported to be remarkably efficient in increasing growth rate and seed yield by 32.6 and 20.4%. In the current research, the well-characterized CaP-U NPs were used for foliar treatment of the finger millet under irrigated and drought conditions. Experimental results revealed that CaP-U NPs significantly improved all the growth-related parameters *viz*., shoot and root length, shoot and root dry weight, shoot and root fresh weight and leaf area of finger millet at both 0.1 and 0.5% concentrations under irrigated and drought conditions. Biochemical parameters such as chlorophyll a, chlorophyll b, total proline content, total phenol content, superoxide dismutase (SOD), peroxidase (POD), ascorbate peroxidase (APX) etc. Also significantly improved by the foliar treatment of CaP-U NPs under irrigated and drought conditions.

The maximum content of chlorophyll a (2.2 and 2.6-fold increase) (**Figure 5A**) and b (2.2 and 4.8-fold increase) (**Figure 5B**) was recorded in CaP-U NPs treated plants, both under irrigated and drought conditions. Previous research has found that by controlling the biosynthesis of PSI, PSII, and LHCs, Cyt b6f, ATP synthase, and photosynthetic enzymes, N supply and allocation within the leaf have a significant impact on photosynthesis. Out of the total nitrogen (N) in leaf cells, 75% is present in the chloroplast (Onoda et al., 2017). About 24% of the N in leaves goes to thylakoids, and 75% of that nitrogen goes to light-harvesting proteins (Zhong et al., 2019). N is a part of the chlorophyll molecule. One molecule of Chl contains 4 molecules of N (Evans and Clarke, 2019), which helps in photosynthesis to absorb sunlight energy, promoting plant growth and grain yield (Li et al., 2008). Drought stress reduced plant photosynthesis by destroying the pigment system. Therefore, retaining green leaves during drought in plants is a significant indicator of drought tolerance.

N has a close relationship with stomatal conductance and/or movement. As the main N source for plants, nitrate could regulate stomatal movements (De Angeli et al., 2006). The leaf analysis from nano fertilizer-treated plots found 17.04% more nitrogen, 16.31% phosphorus, and 67.50% potassium compared to the control; the total chlorophyll content rose to 30.68%, and the net rate of photosynthesis increased to 71.7% (Ha et al., 2019). The number of leaves, plant height, and leaf area of the coffee seedlings under greenhouse conditions were also improved by the use of Chitosan-based NPK nano fertilizer (Ha et al., 2019). In *Sesame indicum* L., the combined application of potassium nano fertilizer and urea could relieve water stress and adverse effects (Mahdavi Khorami et al., 2020). The maximum shoot length was recorded in CaP-U NP at 0.5% with 1.4- and 1.7-fold increase (**Figure 3A**), while maximum root length was also recorded in CaP-U NP at 0.5% with 1.9 and 2-fold increase (**Figure 3B**), respectively. Nitrogen differentially regulates cell elongation and division (Luo et al., 2021). Generally, nitrogen-efficient plants developed a strong root system, which has large root biomass, root volume, root absorption surface area, root active absorption area and a high root oxidation capacity (Xiong et al., 2021). The deposition of lignin and suberin in the roots is controlled by N (Gao et al., 2017). In a study on *C. sativus*, foliar application of CaP-U NPs provided approximately a three-fold increase in the shoot length, as compared to the control, as well as increased nitrogen (N), calcium (Ca) and phosphorus (P) accumulation in both root and shoot (Feil et al., 2021). In wheat crops, foliar application of the chitosan nanoparticles loaded with nitrogen, phosphorus and potassium (NPK) increased shoot length and grain yield substantially (Abdel-Aziz et al., 2016). The leaf area of the plants was significantly impacted under drought conditions. The maximum leaf area was recorded in CaP-U NP at 0.5% with 1.4 and 2.3-fold increases respectively (**Figure 3C**). The maximum plant fresh weight was recorded in CaP-U NP at 0.5% with 1.6 and 2.3-fold increases (**Figures 4A, B**), while the dry biomass of plants decreased due to severe water stress. The maximum plant dry weight was recorded in CaP-U NP at 0.5% with 3.3 and 2.8-fold increases (**Figures 4C, D**) respectively, as compared to control, under irrigated and drought conditions. Pradhan et al. (2021) synthesized Urea Hydroxyapitide (UH) NPs and tested them only for the germination of rice seed (IR-36) and observed that UH NPs substantially increase seedling growth, fresh weight and dry weight of treatments compared to control. Applying CaP-U NPs to *Triticum durum* Desf., significantly increased the fresh and dry weight of the shoot (Ramírez-Rodríguez et al., 2020b). Rice plants fertilized with exogenous urea–chitosan nanohybrid (i.e., 500 mg/L) + 60% classical urea, significantly enhanced the growth and yield-related traits (Elshayb et al., 2022). In *Sorghum bicolor* (L.) Moench, application of the calcium nitrate-gelatin (CNG) coated urea showed maximum dry matter accumulation, high average plant chlorophyll content and apparent nitrogen recovery (ANR) of 71.14% in shoot and 4.5% in roots, respectively (Khan et al., 2021). In *Solanum tuberosum* L., application of the nano-tri combination (N+Mo+B) increased chlorophyll content, the yield of the dry vegetative part, starch content, total protein and ascorbic acid (Al-juthery and Al-Maamouri, 2020). In hydroponics also, *Cucumis sativus* L. supplemented with urea provides a maximum growth of root-shoot length and biomass of the plant (Carmona et al., 2021). In addition, *Pisum sativum* L. treated with SiO_2_ NPs significantly increased their relative water content by 29% and specific leaf area by 17% compared to the non-treated control (Sutulienė et al., 2021). Applying the Chitosan nanoparticles in *Zea mays* significantly enhanced the plant height, leaf area, number of leaves and concentration of organic acids regulators of stress tolerance mechanisms (Khati et al., 2017).

During water scarcity in plants, the activity of the osmoprotectants is increased, which regulates the osmotic potential and increases the stability of membranes and metabolic enzymes of the cells by ROS scavenging (Ahanger et al., 2014). Many researchers suggested a positive correlation between proline accumulation and plant stress. Proline, an amino acid, plays a significant role in alleviating various stress conditions in plants. Besides acting as an excellent osmolyte, proline plays three major roles during stress, i.e., as a metal chelator, an antioxidative defense molecule, and a signaling molecule (Ghosh et al., 2022). Research suggested that proline is 300 times more soluble in water than other amino acids and thus acts as a comparatively non-toxic osmolyte. Nitrogen deficiency in *Phaseolus vulgaris* has declined the proline level by stimulating proline dehydrogenase. However, the proline level was raised under adequate nitrogen due to the activation of ornithine δ-aminotransferase (Sánchez et al., 2002). Proline helps to maintain the structural integrity of the plant cell, as well as the scavenging of reactive oxygen species (ROS) under drought stress. The maximum total proline content was recorded in CaP-U NP at 0.5% with 1.5-fold increase (**Figure 5C**) and total phenol with a 1.6-fold rise (**Figure 5D**) compared to control under drought conditions. No significant changes were observed between control and treated plants under irrigated conditions. The regulation of antioxidant enzymatic activity is a natural response of plants to oxidative stress caused by various external biotic and abiotic stress factors (Mohammadi et al., 2021). To deal with oxidative damage, plants have evolved an excellent defensive strategy of antioxidant enzyme activities such as SOD, POD, CAT, and APX (Xie et al., 2019). SOD catalyzes superoxide (O^-^) elimination by dismutation into oxygen (O_2_) and hydrogen peroxide (H_2_O_2_). The plant’s ability to cope with drought stress is associated with low MDA and H_2_O_2_. Therefore, under drought conditions, the minimum MDA and H_2_O_2_ content were recorded (**Figures 7A, B**) in CaP-U NP-treated plants at 0.5% as well as significantly improved SOD (1.9-fold) (**Figure 6A**), POD (1.4-fold) (**Figure 6B**) and APX (3.3-fold) (**Figure 6C**) activity under drought conditions compared to the control. Here, finger millet plants treated with CaP-U NPs showed a good resistance system to alleviate the damage caused by oxidative stress. The enhanced antioxidant enzymatic activities such as SOD, POD and CAT and reduction in MDA and H_2_O_2_ content indicated an increased redox defense system in response to drought stress. N fertilizer significantly increases leaf superoxide dismutase (SOD) and peroxidase (POD) activities of rice (Jalloh et al., 2009) growing under Cd stress and maize under water stress conditions (Zhang et al., 2007; Ahmad et al., 2022). Foliar application of Chitosan NPs under greenhouse conditions enhanced enzyme activity such as chitosanase, peroxidase and polyphenol oxidase (Sathiyabama and Manikandan, 2021). Chitosan (CHNPs) nanoparticles enhanced phenylalanine ammonia-lyase (PAL), peroxidase (POX), polyphenol oxidase (PPO), catalase (CAT) and β-1, 3 glucanase (GLU) activity in tomato during bacterial wilt infection (Narasimhamurthy et al., 2022). The foliar application of Fullerenol Nanoparticles reduced drought impact by increasing APX in sugarbeets (*Beta vulgaris* L.) (Borisev et al., 2016). During stress conditions, ascorbic acid (AA) acts as a reducing agent, which reduces H_2_O_2_ to H_2_O and dehydroascorbate (DHA) in chloroplasts and cytosols, respectively (Sharma and Dubey, 2004). The cytosolic APX plays a crucial role in protecting plants from drought and heat stress (Koussevitzky et al., 2008). A heme-containing enzyme, GPX removes excess H_2_O_2_ in cytosol and vacuole (Sreenivasulu et al., 2004). The foliar application of chitosan nanoparticles (Cs NPs) on *L. iberica*. during water stress conditions provided the increased activity of superoxide dismutase (SOD), ascorbate peroxidase (APX) and peroxidase (POD) (Javanmard et al., 2022). Foliar and soil treatment of Chitosan NPs (30, 60, and 90 ppm)increased the proline level, catalase (CAT), and superoxide dismutase (SOD) activity during drought stress conditions in *Hordeum vulgare* L. (Behboudi et al., 2018). Si NPs alleviate the stress in finger millet by up-regulating the activity of antioxidant enzymes like APX, CAT, SOD and GPX (Mundada et al., 2021). The foliar application of Fullerene nanoparticles reduced drought impact by decreasing MDA in sugar beets (Borisev et al., 2016). Application of CaP-U NPs significantly improved morphological and physiological traits more compared to similar or higher doses of bulk urea. This improvement in the plants could be linked to the higher N availability from the CaP-U NPs treatment, as compared to the control. Foliar application of CaP-U NPs on finger millet showed positive effects in terms of improved leaf relative water content (LRWC), stomatal conductance, chlorophyll contents, photosynthetic rate, nitrogen assimilation, increased carbohydrate production and N metabolism under drought stress.

## 6 Conclusion

The present study aimed to synthesize and characterize CaP-Urea NPs to assess their potential role in plant growth promotion and defense activation in finger millet under drought conditions. Color changes in visual observation and morphology of NPs by FE-SEM, HR-TEM, and XRD analysis confirmed the synthesized nanoparticles as CaP-U NPs. A broader and valuable outcome of the present work is that a lower dose of CaP-U NPs seems superior in enhancing crop growth, relative to a higher bulk urea dose. Foliar application of the CaP-U NPs increased plant growth indices and activated plant defense under adverse climatic conditions compared to urea as bulk application. Furthermore, CaP-U NPs at 0.5 and 0.1% were found to be slightly more effective in growth indices and defense activation. This investigation demonstrates the roles of nanotechnology in agriculture, one of which is to minimize the input of chemicals into the environment while sustaining crop growth. In future, further studies on yield and nutritional quality improvement using these novel nano-particles under field studies challenged with environmental stresses would provide a deeper understanding of the overall field performance of CaP-U NPs and their potential for commercialization as nano-fertilizer.

## Data availability statement

The original contributions presented in the study are included in the article/supplementary materials. Further inquiries can be directed to the corresponding author.

## Author contributions

DM: conceptualization, methodology, and writing and original draft preparation. PC: supervised the research work and reviewed and edited the manuscript and provided inputs for framing of the manuscript. MC: provided technical assistance and editing of the manuscript. VU: reviewing of the manuscript. JS: provided technical assistance in XRD analysis. All authors contributed to the article and approved the submitted version.

## References

[B1] Abdelaal K. AlKahtani M. Attia K. Hafez Y. Király L. Künstler A. (2021). The role of plant growth-promoting bacteria in alleviating the adverse effects of drought on plants. Biology 10 (6), 1–23. doi: 10.3390/biology10060520 PMC8230635 34207963

[B2] Abdel-Aziz H. M. Hasaneen M. N. Omer A. M. (2016). Nano chitosan-NPK fertilizer enhances the growth and productivity of wheat plants grown in sandy soil. Span. J. Agric. Res. 14 (1), 1–9. doi: 10.5424/sjar/2016141-8205

[B3] Abd El Galil A. El-Salehein A. El Hamady M. (2021). Effect of foliar spray with urea and dry yeast extract on head lettuce production. J. Product. Dev. 26 (4), 1071–1086. doi: 10.21608/jpd.2021.211864

[B4] Ahanger M. A. Tyagi S. R. Wani M. R. Ahmad P. (2014). “Drought tolerance: role of organic osmolytes, growth regulators, and mineral nutrients,” in Physiological mechanisms and adaptation strategies in plants under changing environment (New York, NY: Springer), 25–55. doi: 10.1007/978-1-4614-8591-9_2

[B5] Ahmad S. Wang G. Y. Muhammad I. Chi Y. X. Zeeshan M. Nasar J.. (2022). Interactive effects of melatonin and nitrogen improve drought tolerance of maize seedlings by regulating growth and physiochemical attributes. Antioxidants 11 (2), 1–17. doi: 10.3390/antiox11020359 PMC8869313 35204247

[B6] Ahmed W. Lingner J. (2020). PRDX1 counteracts catastrophic telomeric cleavage events that are triggered by DNA repair activities post oxidative damage. Cell Rep. 33 (5), 1–13. doi: 10.1016/j.celrep.2020.108347 33147465

[B7] Alexieva V. Sergiev I. Mapelli S. Karanov E. (2001). The effect of drought and ultraviolet radiation on growth and stress markers in pea and wheat. Plant Cell Environ. 24 (12), 1337–1344. doi: 10.1046/j.1365-3040.2001.00778.x

[B8] Al-juthery H. W. A. Al-Maamouri E.H. O. (2020). Effect of urea and nano-nitrogen fertigation and foliar application of nano-boron and molybdenum on some growth and yield parameters of potato. Al-Qadisiyah J. Agric. Sci. 10 (1), 253–263. doi: 10.33794/qjas.2020.167074

[B9] Arnon D. I. (1949). Copper enzymes in isolated chloroplasts. polyphenoloxidase in *Beta vulgaris* . Plant Physiol. 24 (1), 1–15. doi: 10.1104/pp.24.1.1 16654194 PMC437905

[B10] Bagheri R. Bashir H. Ahmad J. Iqbal M. Qureshi M. I. (2015). Spinach *(Spinacia oleracea* l.) modulates its proteome differentially in response to salinity, cadmium and their combination stress. Plant Physiol. Biochem. 97, 235–245. doi: 10.1016/j.plaphy.2015.10.012 26497449

[B11] Bates L. S. Waldren R. P. Teare I. D. (1973). Rapid determination of free proline for water-stress studies. Plant Soil. 39 (1), 205–207. doi: 10.1007/BF00018060

[B12] Beauchamp C. Fridovich I. (1971). Superoxide dismutase: improved assays and an assay applicable to acrylamide gels. Anal. Biochem. 44 (1), 276–287. doi: 10.1016/0003-2697(71)90370-8 4943714

[B13] Behboudi F. TahmasebiSarvestani Z. Kassaee M. Z. ModaresSanavi S. A. M. Sorooshzadeh A. Ahmadi S. B. (2018). Evaluation of chitosan nanoparticles effects on yield and yield components of barley *(Hordeum vulgare* l.) under late season drought stress. J. Water Environ. Nanotechnol. 3 (1), 22–39. doi: 10.22090/jwent.2018.01.003

[B14] Ben-Jabeur M. Chamekh Z. Jallouli S. Ayadi S. Serret M. D. Araus J. L.. (2022). Comparative effect of seed treatment with thyme essential oil and paraburkholderiaphytofirmans on growth, photosynthetic capacity, grain yield, δ15N and δ13C of durum wheat under drought and heat stress. Ann. Appl. Biol. 181 (1), 58–69. doi: 10.1111/aab.12754

[B15] Bhavani P. Prakash S. S. Harinikumar K. M. Thimmegowda M. N. Benherlal P. S. Yoganand S. B. (2020). Performance of slow release hydroxyapatite coated urea nanofertilizer on aerobic paddy. Int. J. Curr. Microbiol. App. Sci. 9 (11), 1320–1330. doi: 10.20546/ijcmas.2020.911.155

[B16] Borisev M. Borisev I. Zupunski M. Arsenov D. Pajević S. Curcic Z.. (2016). Drought impact is alleviated in sugar beets *(Beta vulgaris* l.) by foliar application of fullerenol nanoparticles. PLoS One 11 (11), 1–20. doi: 10.1371/journal.pone.0166248 PMC5104475 27832171

[B17] Carmona F. J. Dal Sasso G. Ramírez-Rodríguez G. B. Pii Y. Delgado-López J. M. Guagliardi A.. (2021). Urea-functionalized amorphous calcium phosphate nanofertilizers: optimizing the synthetic strategy towards environmental sustainability and manufacturing costs. Sci. Rep. 11 (1), 1–14. doi: 10.1038/s41598-021-83048-9 33564033 PMC7873063

[B18] Caverzan A. Casassola A. Brammer S. P. (2016). Antioxidant responses of wheat plants under stress. Genet. Mol. Biol. 39, 1–6. doi: 10.1590/1678-4685-GMB-2015-0109 27007891 PMC4807390

[B19] Cesco S. Tolotti A. Nadalini S. Rizzi S. Valentinuzzi F. Mimmo T.. (2020). *Plasmoparaviticola* Infection affects mineral elements allocation and distribution in *Vitis vinifera* leaves. Sci. Rep. 10 (1), 1–18. doi: 10.1038/s41598-020-75990-x 33127977 PMC7603344

[B20] Chandra A. K. Pandey D. Tiwari A. Gururani K. Agarwal A. Dhasmana A.. (2021). Metal based nanoparticles trigger the differential expression of key regulatory genes which regulate iron and zinc homeostasis mechanism in finger millet. J. Cereal Sci. 100, 1–14. doi: 10.1016/j.jcs.2021.103235

[B21] Chang Z. Liu Y. Dong H. Teng K. Han L. Zhang X. (2016). Effects of cytokinin and nitrogen on drought tolerance of creeping bentgrass. PLoS One 11 (4), 1–19. doi: 10.1371/journal.pone.0154005 PMC4839601 27099963

[B22] Chethan S. Malleshi N. G. (2007). Finger millet polyphenols: optimization of extraction and the effect of pH on their stability. Food Chem. 105 (2), 862–870. doi: 10.1016/j.foodchem.2007.02.012

[B23] Chhipa H. (2017). Nanofertilizers and nanopesticides for agriculture. Environ. Chem. Lett. 15 (1), 15–22. doi: 10.1007/s10311-016-0600-4

[B24] Chitara M. K. Chetan Keswani C. K. Kartikay Bisen K. B. Vivek Singh V. S. Singh S. P. Sarma B. K.. (2017). “Improving crop performance under heat stress using thermotolerant agriculturally important microorganisms,” in Advances in PGPR research (Wallingford UK: CABI), 296–305. doi: 10.1079/9781786390325.0296

[B25] De Angeli A. Monachello D. Ephritikhine G. Frachisse J. M. Thomine S. Gambale F.. (2006). The nitrate/proton antiporter AtCLCa mediates nitrate accumulation in plant vacuoles. Nature 442 (7105), 939–942. doi: 10.1038/nature05013 16878138

[B26] Deblonde P. M. K. Ledent J. F. (2001). Effects of moderate drought conditions on green leaf number, stem height, leaf length and tuber yield of potato cultivars. Eur. J. Agron. 14 (1), 31–41. doi: 10.1016/S1161-0301(00)00081-2

[B27] Ding L. Lu Z. Gao L. Guo S. Shen Q. (2018). Is nitrogen a key determinant of water transport and photosynthesis in higher plants upon drought stress? Front. Plant Sci. 9. doi: 10.3389/fpls.2018.01143 PMC6113670 30186291

[B28] Eichert T. Kurtz A. Steiner U. Goldbach H. E. (2008). Size exclusion limits and lateral heterogeneity of the stomatal foliar uptake pathway for aqueous solutes and water-suspended nanoparticles. Physiol. Plant 134 (1), 151–160. doi: 10.1111/j.1399-3054.2008.01135.x 18494856

[B29] Elshayb O. M. Nada A. M. Farroh K. Y. AL-Huqail A. A. Aljabri M. Binothman N.. (2022). Utilizing urea–chitosan nanohybrid for minimizing synthetic urea application and maximizing *Oryza sativa* l. productivity and n uptake. Agriculture 12 (7), 1–15. doi: 10.3390/agriculture12070944

[B30] Evans J. R. Clarke V. C. (2019). The nitrogen cost of photosynthesis. J. Exp. Bot. 70 (1), 7–15. doi: 10.1093/jxb/ery366 30357381

[B31] Feil S. B. Rodegher G. Gaiotti F. Zuluaga M. Y. A. Carmona F. J. Masciocchi N.. (2021). Physiological and molecular investigation of urea uptake dynamics in *Cucumis sativus* l. plants fertilized with urea-doped amorphous calcium phosphate nanoparticles. Front. Plant Sci. 12. doi: 10.3389/fpls.2021.745581 PMC8688946 34950161

[B32] Gaiotti F. Lucchetta M. Rodegher G. Lorenzoni D. Longo E. Boselli E.. (2021). Urea-doped calcium phosphate nanoparticles as sustainable nitrogen nanofertilizers for viticulture: implications on yield and quality of pinot gris grapevines. Agronomy 11 (6), 1–16. doi: 10.3390/agronomy11061026

[B33] Gao C. Ding L. Li Y. Chen Y. Zhu J. Gu M.. (2017). Nitrate increases ethylene production and aerenchyma formation in roots of lowland rice plants under water stress. Funct. Plant Biol. 44 (4), 430–442. doi: 10.1071/FP16258 32480576

[B34] Ghani M. I. Saleem S. Rather S. A. Rehmani M. S. Alamri S. Rajput V. D.. (2022). Foliar application of zinc oxide nanoparticles: an effective strategy to mitigate drought stress in cucumber seedling by modulating antioxidant defense system and osmolytes accumulation. Chemosphere 289, 133202. doi: 10.1016/j.chemosphere.2021.133202 34890613

[B35] Ghosh U. K. Islam M. N. Siddiqui M. N. Cao X. Khan M. A. R. (2022). Proline, a multifaceted signalling molecule in plant responses to abiotic stress: understanding the physiological mechanisms. Plant Biol. 24 (2), 227–239. doi: 10.1111/plb.13363 34796604

[B36] Giroto A. S. Guimarães G. G. Foschini M. Ribeiro C. (2017). Role of slow-release nanocomposite fertilizers on nitrogen and phosphate availability in soil. Sci. Rep. 7 (1), 1–11. doi: 10.1038/srep46032 28406141 PMC5390257

[B37] Gui Y. W. Sheteiwy M. S. Zhu S. G. Batool A. Xiong Y. C. (2021a). Differentiate effects of non-hydraulic and hydraulic root signaling on yield and water use efficiency in diploid and tetraploid wheat under drought stress. Environ. Exp. Bot. 181, 1–18. doi: 10.1016/j.envexpbot.2020.104287

[B38] Gui Y. Sheteiwy M. S. Zhu S. Zhu L. Batool A. Jia T.. (2021b). Differentiate responses of tetraploid and hexaploid wheat (Triticum aestivum l.) to moderate and severe drought stress: a cue of wheat domestication. Plant Signal. Behav. 16 (1), 1–13. doi: 10.1080/15592324.2020.1839710 PMC7781840 33126814

[B39] Gunning B. E. Steer M. W. (1996). Plant cell biology: structure and function. Jones Bartlett Learn., 1–5.

[B40] Ha N. M. C. Nguyen T. H. Wang S. L. Nguyen A. D. (2019). Preparation of NPK nanofertilizer based on chitosan nanoparticles and its effect on biophysical characteristics and growth of coffee in green house. Res. Chem. Intermed. 45, 51–63. doi: 10.1007/s11164-018-3630-7

[B41] Hadimani N. A. Malleshi N. G. (1993). Studies on milling, physico-chemical properties, nutrient composition and dietary fibre content of millets. J. Food Sci. Technol. 30 (1), 17–20.

[B42] Handayati W. Sihombing D. (2019). “Study of NPK fertilizer effect on sunflower growth and yield,” in AIP conference proceedings, vol. 2120. (AIP Publishing LLC), 030031. doi: 10.1063/1.5115635

[B43] Heath R. L. Packer L. (1968). Photoperoxidation in isolated chloroplasts: i. kinetics and stoichiometry of fatty acid peroxidation. Arch. Biochem. Biophys. 125 (1), 189–198. doi: 10.1016/0003-9861(68)90654-1 5655425

[B44] Hemati A. Moghiseh E. Amirifar A. Mofidi-Chelan M. AsgariLajayer B. (2022). “Physiological effects of drought stress in plants,” in Plant stress mitigators (Singapore: Springer), 113–124. doi: 10.1007/978-981-16-7759-5_6

[B45] Ho M. D. Rosas J. C. Brown K. M. Lynch J. P. (2005). Root architectural tradeoffs for water and phosphorus acquisition. Funct. Plant Biol. 32 (8), 737–748. doi: 10.1071/FP05043 32689171

[B46] Hsiao T. C. Xu L. K. (2000). Sensitivity of growth of roots versus leaves to water stress: biophysical analysis and relation to water transport. J. Exp. Bot. 51 (350), 1595–1616. doi: 10.1093/jexbot/51.350.1595 11006310

[B47] Hussain M. Z. Bhardwaj A. K. Basso B. Robertson G. P. Hamilton S. K. (2019). Nitrate leaching from continuous corn, perennial grasses, and poplar in the US Midwest. J. Environ. Qual. 48 (6), 1849–1855. doi: 10.2134/jeq2019.04.0156

[B48] Jalloh M. A. Chen J. Zhen F. Zhang G. (2009). Effect of different n fertilizer forms on antioxidant capacity and grain yield of rice growing under cd stress. J. Hazard. Mater. 162 (2-3), 1081–1085. doi: 10.1016/j.jhazmat.2008.05.146 18603363

[B49] Javanmard A. Ashrafi M. Morshedloo M. R. Machiani M. A. Rasouli F. Maggi F. (2022). Optimizing phytochemical and physiological characteristics of balangu *(Lallemantiaiberica*) by foliar application of chitosan nanoparticles and myco-root inoculation under water supply restrictions. Horticulturae 8 (8), 1–17. doi: 10.3390/horticulturae8080695

[B50] Ju Y. L. Yue X. F. Zhao X. F. Zhao H. Fang Y. L. (2018). Physiological, micro-morphological and metabolomic analysis of grapevine (Vitis vinifera l.) leaf of plants under water stress. Plant Physiol. Biochem. 130, 501–510. doi: 10.1016/j.plaphy.2018.07.036 30096685

[B51] Kar M. Mishra D. (1975). Inorganic pyrophosphatase activity during rice leaf senescence. Canad. J. Bot. 53 (5), 503–511. doi: 10.1139/b75-061

[B52] Khan O. Niazi M. B. K. Shah G. A. Hazafa A. Jahan Z. Sadiq M.. (2021). Green synthesis and evaluation of calcium-based nanocomposites fertilizers: a way forward to sustainable agricultural. J. Saudi Soc Agric. Sci. 20 (8), 519–529. doi: 10.1016/j.jssas.2021.06.005

[B53] Khati P. Chaudhary P. Gangola S. Bhatt P. Sharma A. (2017). Nanochitosan supports growth of zea mays and also maintains soil health following growth. 3 Biotech. 7 (1), 1–9. doi: 10.1007/s13205-017-0668-y PMC5429309 28500403

[B54] Koch G. Rolland G. Dauzat M. Bédiée A. Baldazzi V. Bertin N.. (2019). Leaf production and expansion: a generalized response to drought stresses from cells to whole leaf biomass–a case study in the tomato compound leaf. Plants 8 (10), 1–17. doi: 10.3390/plants8100409 PMC6843756 31614737

[B55] Kopittke P. M. Lombi E. Wang P. Schjoerring J. K. Husted S. (2019). Nanomaterials as fertilizers for improving plant mineral nutrition and environmental outcomes. Environ. Sci. Nano 6 (12), 3513–3524. doi: 10.1039/C9EN00971J

[B56] Kottegoda N. Sandaruwan C. Priyadarshana G. Siriwardhana A. Rathnayake U. A. Berugoda Arachchige D. M.. (2017). Urea-hydroxyapatite nanohybrids for slow release of nitrogen. ACS nano 11 (2), 1214–1221. doi: 10.1021/acsnano.6b07781 28121129

[B57] Koussevitzky S. Suzuki N. Huntington S. Armijo L. Sha W. Cortes D.. (2008). Ascorbate peroxidase 1 plays a key role in the response of *Arabidopsis thaliana* to stress combination. J. Biol. Chem. 283 (49), 34197–34203. doi: 10.1074/jbc.M806337200 18852264 PMC2590703

[B58] Krishnan R. Gnanaseelan C. Sanjay J. Swapna P. Dhara C. Sabin T. P.. (2020). “Introduction to climate change over the Indian region,” in Assessment of climate change over the Indian region (Singapore: Springer), 1–20. doi: 10.1007/978-981-15-4327-2_13

[B59] Kumar A. Gaur V. S. Goel A. Gupta A. K. (2015). *De novo* Assembly and characterization of developing spikes transcriptome of finger millet *(Eleusine coracana)*: a minor crop having nutraceutical properties. Plant Mol. Biol. Rep. 33 (4), 905–922. doi: 10.1007/s11105-014-0802-5

[B60] Kumar A. Singh K. Verma P. Singh O. Panwar A. Singh T.. (2022). Effect of nitrogen and zinc nanofertilizer with the organic farming practices on cereal and oil seed crops. Sci. Rep. 12 (1), 1–7. doi: 10.1038/s41598-022-10843-3 35484376 PMC9050747

[B61] Kumari A. Bhinda M. S. Sharma S. Chitara M. K. Debnath A. Maharana C. (2021). ROS regulation mechanism for mitigation of abiotic stress in plants. Reactive Oxygen Species 28, 197–238. doi: 10.5772/intechopen.99845

[B62] Ladha J. K. Pathak H. Krupnik T. J. Six J. van Kessel C. (2005). Efficiency of fertilizer nitrogen in cereal production: retrospects and prospects. Adv. Agron. 87, 85–156. doi: 10.1016/S0065-2113(05)87003-8

[B63] Lesk C. Rowhani P. Ramankutty N. (2016). Influence of extreme weather disasters on global crop production. Nature 529 (7584), 84–99. doi: 10.1038/nature16467 26738594

[B64] Li Y. Horsman M. Wang B. Wu N. Lan C. Q. (2008). Effects of nitrogen sources on cell growth and lipid accumulation of green alga neochlorisoleoabundans. Appl. Microbiol. Biotechnol. 81 (4), 629–636. doi: 10.1007/s00253-008-1681-1 18795284

[B65] Li G. Tillard P. Gojon A. Maurel C. (2016). Dual regulation of root hydraulic conductivity and plasma membrane aquaporins by plant nitrate accumulation and high-affinity nitrate transporter NRT2. 1. Plant Cell Physiol. 57 (4), 733–742. doi: 10.1093/pcp/pcw022 26823528

[B66] Liu R. Lal R. (2015). Potentials of engineered nanoparticles as fertilizers for increasing agronomic productions. Sci. Total Environ. 514, 131–139. doi: 10.1016/j.scitotenv.2015.01.104 25659311

[B67] Luo Y. Peng Q. Li K. Gong Y. Liu Y. Han W. (2021). Patterns of nitrogen and phosphorus stoichiometry among leaf, stem and root of desert plants and responses to climate and soil factors in xinjiang, China. Catena 199, 1–25. doi: 10.1016/j.catena.2020.105100

[B68] Mahdavi Khorami A. Masoud Sinaki J. Amini Dehaghi M. Rezvan S. Damavandi A. (2020). Sesame *(Sesame indicum* l.) biochemical and physiological responses as affected by applying chemical, biological, and nano-fertilizers in field water stress conditions. J. Plant Nutr. 43 (3), 456–475. doi: 10.1080/01904167.2019.1683189

[B69] Mahil E. I. T. Kumar B. A. (2019). Foliar application of nanofertilizers in agricultural crops–a review. J. Farm Sci. 32 (3), 239–249.

[B70] Mahmoud A. W. M. Swaefy H. M. (2020). Comparison between commercial and nano NPK in presence of nano zeolite on sage plant yield and its components under water stress. Agriculture 66 (1), 24–39. doi: 10.2478/agri-2020-0003

[B71] Manikandan A. Subramanian K. S. (2016). Evaluation of zeolite based nitrogen nano-fertilizers on maize growth, yield and quality on inceptisols and alfisols. Int. J. Plant Soil Sci. 9 (4), 1–9. doi: 10.9734/IJPSS/2016/22103

[B72] Mohammadi M. A. Cheng Y. Aslam M. Jakada B. H. Wai M. H. Ye K.. (2021). ROS and oxidative response systems in plants under biotic and abiotic stresses: revisiting the crucial role of phosphite triggered plants defense response. Front. Microbiol. 12. doi: 10.3389/fmicb.2021.631318 PMC8281016 34276579

[B73] Mohammadian R. Moghaddam M. Rahimian H. Sadeghian S. Y. (2005). Effect of early season drought stress on growth characteristics of sugar beet genotypes. Turk. J. Agric. For. 29 (5), 357–368.

[B74] Mohammadkhani N. Heidari R. (2008). Effects of drought stress on soluble proteins in two maize varieties. Turk. J. Biol. 32 (1), 23–30.

[B75] Morgan T. T. Muddana H. S. Altinoglu E. I. Rouse S. M. Tabakovic A. Tabouillot T.. (2008). Encapsulation of organic molecules in calcium phosphate nanocomposite particles for intracellular imaging and drug delivery. Nano Lett. 8 (12), 4108–4115. doi: 10.1021/nl8019888 19367837 PMC3267632

[B76] Mundada P. S. Ahire M. L. Umdale S. D. Barmukh R. B. Nikam T. D. Pable A. A.. (2021). Characterization of influx and efflux silicon transporters and understanding their role in the osmotic stress tolerance in finger millet *(Eleusinecoracana* (L.) gaertn.). Plant Physiol. Biochem. 162, 677–689. doi: 10.1016/j.plaphy.2021.03.033 33780741

[B77] Naeem M. Naeem M. S. Ahmad R. Ihsan M. Z. Ashraf M. Y. Hussain Y.. (2018). Foliar calcium spray confers drought stress tolerance in maize *via* modulation of plant growth, water relations, proline content and hydrogen peroxide activity. Arch. Agron. Soil Sci. 64 (1), 116–131. doi: 10.1080/03650340.2017.1327713

[B78] Nakano Y. Asada K. (1981). Hydrogen peroxide is scavenged by ascorbate-specific peroxidase in spinach chloroplasts. Plant Cell Physiol. 22 (5), 867–880. doi: 10.1093/oxfordjournals.pcp.a076232

[B79] Narasimhamurthy K. Udayashankar A. C. De Britto S. Lavanya S. N. Abdelrahman M. Soumya K.. (2022). Chitosan and chitosan-derived nanoparticles modulate enhanced immune response in tomato against bacterial wilt disease. Int. J. Biol. Macromol. 220, 223–237. doi: 10.1016/j.ijbiomac.2022.08.054 35970370

[B80] Ning D. Qin A. Liu Z. Duan A. Xiao J. Zhang J.. (2020). Silicon-mediated physiological and agronomic responses of maize to drought stress imposed at the vegetative and reproductive stages. Agronomy 10 (8), 1–19. doi: 10.3390/agronomy10081136

[B81] Onoda Y. Wright I. J. Evans J. R. Hikosaka K. Kitajima K. Niinemets Ü.. (2017). Physiological and structural tradeoffs underlying the leaf economics spectrum. New Phytol. 214 (4), 1447–1463. doi: 10.1111/nph.14496 28295374

[B82] Orimoloye I. R. Belle J. A. Orimoloye Y. M. Olusola A. O. Ololade O. O. (2022). Drought: a common environmental disaster. Atmosphere 13 (1), 1–21. doi: 10.3390/atmos13010111

[B83] Ortiz-Gómez I. Ramírez-Rodríguez G. B. Capitán-Vallvey L. F. Salinas-Castillo A. Delgado-López J. M. (2020). Highly stable luminescent europium-doped calcium phosphate nanoparticles for creatinine quantification. Colloids Surf. B: Biointerfaces. 196, 1–8. doi: 10.1016/j.colsurfb.2020.111337 32949922

[B84] Oumarou Abdoulaye A. Lu H. Zhu Y. Alhaj Hamoud Y. Sheteiwy M. (2019). The global trend of the net irrigation water requirement of maize from 1960 to 2050. Climate 7 (10), 1–19. doi: 10.3390/cli7100124

[B85] Pan Y. She D. Chen X. Xia Y. Timm L. C. (2021). Elevation of biochar application as regulator on denitrification/NH3 volatilization in saline soils. Environ. Sci. pollut. Res. 28 (31), 41712–41725. doi: 10.1007/s11356-021-13562-w 33786768

[B86] Passioura J. B. (2002). Soil conditions and plant growth. Plant Cell Environ. 25 (2), 311–318. doi: 10.1046/j.0016-8025.2001.00802.x 11841672

[B87] Pradhan S. Durgam M. Mailapalli D. R. (2021). Urea loaded hydroxyapatite nanocarrier for efficient delivery of plant nutrients in rice. Arch. Agron. Soil Sci. 67 (3), 371–382. doi: 10.1080/03650340.2020.1732940

[B88] Rajonee A. A. Nigar F. Ahmed S. Imamul Huq S. M. (2016). Synthesis of nitrogen nano fertilizer and its efficacy. Can. J. Pur. Appl. Sci. 10, 3913–3919.

[B89] Ramírez-Rodríguez G. B. Dal Sasso G. Carmona F. J. Miguel-Rojas C. Pérez-de-Luque A. Masciocchi N.. (2020a). Engineering biomimetic calcium phosphate nanoparticles: a green synthesis of slow-release multinutrient (NPK) nanofertilizers. ACS Appl. Bio Mater. 3 (3), 1344–1353. doi: 10.1021/acsabm.9b00937 35021628

[B90] Ramírez-Rodríguez G. B. Miguel-Rojas C. Montanha G. S. Carmona F. J. Dal Sasso G. Sillero J. C.. (2020b). Reducing nitrogen dosage in triticum durum plants with urea-doped nanofertilizers. J. Nanomater. 10 (6), 1–16. doi: 10.3390/nano10061043 PMC7353301 32486000

[B91] Ren J. Sun L. N. Zhang Q. Y. Song X. S. (2016). Drought tolerance is correlated with the activity of antioxidant enzymes in cerasus humilis seedlings. BioMed. Res. Int. 7, 1–9. doi: 10.1155/2016/9851095 PMC4800087 27047966

[B92] Ren B. Wang M. Chen Y. Sun G. Li Y. Shen Q.. (2015). Water absorption is affected by the nitrogen supply to rice plants. Plant Soil 396 (1), 397–410. doi: 10.1007/s11104-015-2603-5

[B93] Rico C. M. Majumdar S. Duarte-Gardea M. Peralta-Videa J. R. Gardea-Torresdey J. L. (2011). Interaction of nanoparticles with edible plants and their possible implications in the food chain. J. Agric. Food Chem. 59 (8), 3485–3498. doi: 10.1021/jf104517j 21405020 PMC3086136

[B94] Sachdev S. Ansari S. A. Ansari M. I. Fujita M. Hasanuzzaman M. (2021). Abiotic stress and reactive oxygen species: generation, signaling, and defense mechanisms. Antioxidants 10 (2), 1–37. doi: 10.3390/antiox10020277 PMC7916865 33670123

[B95] Sánchez E. Garcia P. C. López-Lefebre L. R. Rivero R. M. Ruiz J. M. Romero L. (2002). Proline metabolism in response to nitrogen deficiency in French bean plants (Phaseolus vulgaris l. cv strike). Plant Growth Regul. 36 (3), 261–265. doi: 10.1023/A:1016583430792

[B96] Sathiyabama M. Manikandan A. (2021). Foliar application of chitosan nanoparticle improves yield, mineral content and boosts innate immunity in finger millet plants. Carbohydr. Polym. 258, 1–8. doi: 10.1016/j.carbpol.2021.117691 33593564

[B97] Seleiman M. F. Al-Suhaibani N. Ali N. Akmal M. Alotaibi M. Refay Y.. (2021). Drought stress impacts on plants and different approaches to alleviate its adverse effects. Plants 10 (2), 1–25. doi: 10.3390/plants10020259 PMC7911879 33525688

[B98] Shakeel A. A. Xiao-yu X. Long-chang W. Muhammad F. S. Chen M. Wang L. (2011). Morphological, physiological and biochemical responses of plants to drought stress. Afr. J. Agric. Res. 6 (9), 2026–2032. doi: 10.5897/AJAR10.027

[B99] Sharma P. Dubey R. S. (2004). Ascorbate peroxidase from rice seedlings: properties of enzyme isoforms, effects of stresses and protective roles of osmolytes. Plant Sci. 167 (3), 541–550. doi: 10.1016/j.plantsci.2004.04.028

[B100] Sharma A. Gupta P. Prabhakar P. K. (2019). Endogenous repair system of oxidative damage of DNA. Curr. Chem. Biol. 13 (2), 110–119. doi: 10.2174/2212796813666190221152908

[B101] Sharma D. Jamra G. Singh U. M. Sood S. Kumar A. (2017). Calcium biofortification: three pronged molecular approaches for dissecting complex trait of calcium nutrition in finger millet *(Eleusinecoracana*) for devising strategies of enrichment of food crops. Front. Plant Sci. 7. doi: 10.3389/fpls.2016.02028 PMC5239788 28144246

[B102] Sharma S. K. Sharma P. K. Mandeewal R. L. Sharma V. Chaudhary R. Pandey R.. (2022). Effect of foliar application of nano-urea under different nitrogen levels on growth and nutrient content of pearl millet (Pennisetum glaucum l.). Int. J. Plant Sci. 34 (20), 149–155. doi: 10.9734/ijpss/2022/v34i2031138

[B103] Sheteiwy M. S. Abd Elgawad H. Xiong Y. C. Macovei A. Brestic M. Skalicky M.. (2021a). Inoculation with bacillus amyloliquefaciens and mycorrhiza confers tolerance to drought stress and improve seed yield and quality of soybean plant. Physiol. Plant 172 (4), 2153–2169. doi: 10.1111/ppl.13454 33964177

[B104] Sheteiwy M. S. Ali D. F. I. Xiong Y. C. Brestic M. Skalicky M. Hamoud Y. A.. (2021b). Physiological and biochemical responses of soybean plants inoculated with arbuscular mycorrhizal fungi and bradyrhizobium under drought stress. BMC Plant Biol. 21 (1), 1–21. doi: 10.1186/s12870-021-02949-z 33888066 PMC8061216

[B105] Sheteiwy M. Ulhassan Z. Qi W. Lu H. AbdElgawad H. Minkina T.. (2022). Association of jasmonic acid priming with multiple defense mechanisms in wheat plants under high salt stress. Front. Plant Sci. 2614. doi: 10.3389/fpls.2022.886862 PMC9429808 36061773

[B106] Sial N. Y. Faheem M. Sial M. A. Roonjho A. R. Muhammad F. Keerio A. A.. (2022). Exotic wheat genotypes response to water-stress conditions. J. Breed. Genet. 54 (2), 297–304. doi: 10.54910/sabrao2022.54.2.8

[B107] Singh P. P. Priyam A. Singh J. Gupta N. (2023). Biologically synthesised urea-based nanomaterial shows enhanced agronomic benefits in maize and rice crops during kharif season. Sci. Hortic. 315, 1–21. doi: 10.1016/j.scienta.2023.111988

[B108] Soliman A. S. Hassan M. Abou-Elella F. Ahmed A. H. El-Feky S. A. (2016). Effect of nano and molecular phosphorus fertilizers on growth and chemical composition of baobab (Adansonia digitata l.). J. Plant Sci. 11 (4), 52–60. doi: 10.3923/jps.2016.52.60

[B109] Song Y. Li J. Liu M. Meng Z. Liu K. Sui N. (2019). Nitrogen increases drought tolerance in maize seedlings. Funct. Plant Biol. 46 (4), 350–359. doi: 10.1071/FP18186 32172744

[B110] Sreenivasulu N. Miranda M. Prakash H. S. Wobus U. Weschke W. (2004). Transcriptome changes in foxtail millet genotypes at high salinity: identification and characterization of a PHGPX gene specifically up-regulated by NaCl in a salt-tolerant line. J. Plant Physiol. 161 (4), 467–477. doi: 10.1078/0176-1617-01112 15128034

[B111] Subramanian K. S. Manikandan A. Thirunavukkarasu M. Rahale C. S. (2015). “Nano-fertilizers for balanced crop nutrition,” in Nanotechnologies in food and agriculture (Cham: Springer), 69–80. doi: 10.1007/978-3-319-14024-7_3

[B112] Sutulienė R. Ragelienė L. Samuolienė G. Brazaitytė A. Urbutis M. Miliauskienė J. (2021). The response of antioxidant system of drought-stressed green pea *(Pisum sativum* l.) affected by watering and foliar spray with silica nanoparticles. Horticulturae 8 (1), 1–15. doi: 10.3390/horticulturae8010035

[B113] Tarafder C. Daizy M. Alam M. M. Ali M. R. Islam M. J. Islam R.. (2020). Formulation of a hybrid nanofertilizer for slow and sustainable release of micronutrients. ACS omega 5 (37), 23960–23966. doi: 10.1021/acsomega.0c03233 32984716 PMC7513328

[B114] Verma A. K. Deepti S. (2016). Abiotic stress and crop improvement: current scenario. Adv. Plants Agric. Res. 4 (4), 345–346. doi: 10.15406/apar.2016.04.00149

[B115] Wang K. Bu T. Cheng Q. Dong L. Su T. Chen Z.. (2021). Two homologous LHY pairs negatively control soybean drought tolerance by repressing the abscisic acid responses. New Phytol. 229 (5), 2660–2675. doi: 10.1111/nph.17019 33095906

[B116] Xie X. He Z. Chen N. Tang Z. Wang Q. Cai Y. (2019). The roles of environmental factors in regulation of oxidative stress in plant. BioMed. Res. Int. 2019, 1–11. doi: 10.1155/2019/9732325 PMC6530150 31205950

[B117] Xiong Q. Hu J. Wei H. Zhang H. Zhu J. (2021). Relationship between plant roots, rhizosphere microorganisms, and nitrogen and its special focus on rice. Agriculture 11 (3), 1–18. doi: 10.3390/agriculture11030234

[B118] Yadav B. Jogawat A. Rahman M. S. Narayan O. P. (2021). Secondary metabolites in the drought stress tolerance of crop plants: a review. Gene Rep. 23, 1–14. doi: 10.1016/j.genrep.2021.101040 33421146

[B119] Zambryski P. (2004). Cell-to-cell transport of proteins and fluorescent tracers *via* plasmodesmata during plant development. Int. J. Cell Biol. 164 (2), 165–168. doi: 10.1083/jcb.200310048 PMC2172327 14734529

[B120] Zhang L. X. Li S. X. Zhang H. Liang Z. S. (2007). Nitrogen rates and water stress effects on production, lipid peroxidation and antioxidative enzyme activities in two maize (Zea mays l.) genotypes. J. Agron. Crop Sci. 193 (6), 387–397. doi: 10.1111/j.1439-037X.2007.00276.x

[B121] Zhang G. St. Clair A. L. Dolezal A. G. Toth A. L. O’Neal M. E. (2022). Can native plants mitigate climate-related forage dearth for honey bees (Hymenoptera: apidae)? J. Econ. Entomol. 115 (1), 1–9. doi: 10.1093/jee/toab202 34850022 PMC8827321

[B122] Zhong C. Cao X. Hu J. Zhu L. Zhang J. Huang J.. (2017). Nitrogen metabolism in adaptation of photosynthesis to water stress in rice grown under different nitrogen levels. Front. Plant Sci. 8. doi: 10.3389/fpls.2017.01079 PMC5481364 28690622

[B123] Zhong C. Jian S. F. Huang J. Jin Q. Y. Cao X. C. (2019). Trade-off of within-leaf nitrogen allocation between photosynthetic nitrogen-use efficiency and water deficit stress acclimation in rice (Oryza sativa l.). Plant Physiol. Biochem. 135, .41–.50. doi: 10.1016/j.plaphy.2018.11.021 30500517

[B124] Zieslin N. Ben Zaken R. (1993). Peroxidase activity and presence of phenolic substances in peduncles of rose flowers. Plant Physiol. Biochem. 31, 3, 333–339.

[B125] Zulfiqar F. Navarro M. Ashraf M. Akram N. A. Munné-Bosch S. (2019). Nanofertilizer use for sustainable agriculture: advantages and limitations. Plant Sci. 289, 1–7. doi: 10.1016/j.plantsci.2019.110270 31623775

